# Supplementary material for: Simulation of NMR spectra at zero and ultralow fields from A to Z – a tribute to Prof. Konstantin L'vovich Ivanov
Source: Magn Reson (Gott). 2023 Apr 11;4(1):87–109. doi: 10.5194/mr-4-87-2023 (PMC11034480; doi:10.5194/mr-4-87-2023)
Supplement: The supplement related to this article is available online at: https://doi.org/10.5194/mr-4-87-2023-supplement. [file mr-4-87-2023-supplement.pdf]

Supplement of Magn. Reson., 4, 87–109, 2023
https://doi.org/10.5194/mr-4-87-2023-supplement
© Author(s) 2023. CC BY 4.0 License.

Open Access
MAGNETIC RESONANCE

Supplement of

Simulation of NMR spectra at zero and ultralow fields from A to Z – a tribute to Prof. Konstantin L'vovich Ivanov

Quentin Stern and Kirill Sheberstov

Correspondence to: Quentin Stern (quentin.stern@protonmail.com)

The copyright of individual parts of the supplement might differ from the article licence.

Contents

S1. Explicit calculation of the eigenbasis for XA_2 spin system
S2. Tests on ideal signals to show the efficiency of the Fourier transform algorithm
S3. Simulation of NMR spectra at zero and ultra-low field vs high field
S4. Simulation of NMR spectra at zero and ultra-low field - Case of XA spin system
S5. Simulation of NMR spectra at zero and ultra-low field - Case of XAn spin system
S6. Analytical expressions for the eigenenergies and transition amplitudes of XAn spin systems at zero field

The codes presented here as PDF files can be downloaded from this link:
https://doi.org/10.5281/zenodo.7758782

S1. Explicit calculation of the eigenbasis for XA_2 spin system

Below we calculate explicitly the states of the eigenbasis for the XA_2 spin system. An arbitrary spin state of the XA_2 spin system can be represented as a linear combination of states obtained by direct products of the individual Zeeman states: $\mathcal{B}_Z^8 = \{\alpha,\beta\} \otimes \{\alpha,\beta\} \otimes \{\alpha,\beta\}$; here, the superscript 8 represents the dimension of the Hilbert space. The goal is to explicitly express the eigenbasis of the *J*-coupling Hamiltonian that governs ZULF evolution in terms of the states of the Zeeman basis $\mathcal{B}_Z^8$. This is achieved by adding up the angular momenta of individual spins taking into account Clebsch-Gordan coefficients to construct the state (see Eq. 46 of the main text). Figure 7 in the main text illustrates two stages of adding up angular momenta for the XA_2 case: A + A and A_2 + X. There are four states with total spin F = 3/2 and four more states with total spin F = 1/2. The full specification of an eigenfunction at zero field requires three quantum numbers: the total spin of all three spins F, its projection m_F, and the total spin of some of the two spins. Observable transitions at ZULF conserve the total spin of the A_2 subsystem (see Eq. 59 in the main text), so it makes sense to consider the eigenfunctions that are characterised by the total spin of A_2 subsystem, F_{A2}. Therefore, three quantum numbers $|F, m, F_{A2}\rangle$ are used to specify all eight eigenstates.

Eq. 46 of the main text gives the expression of the eigenfunctions of the *J*-coupling Hamiltonian in terms of the Zeeman states and the Clebsch-Gordan coefficients for a pair of spins. Because we are considering three spins, we need to use Eq. 46 twice (for each addition operation) yielding the expression:

$$|F, m, F_{A2}\rangle \;=\; \sum_{m_I, m_S} C^{F_{A2}, m_{A2}}_{I_{a1}, m_{a1}; I_{a2}, m_{a2}} C^{F,m}_{F_{A2}, m_{A2}; S, m_S} |I_{a1}, m_{a1}, I_{a2}, m_{a2}\, S, m_S\,\rangle, \qquad \text{Eq S1}$$

Here, I_{a1} and m_{a1} are the total spin and its projection of the first spin A, respectively, likewise I_{a2} and m_{a2} are those for the second spin A, and S and m_S are the total spin and its projection for spin X, respectively. As an example, let us consider the $|3/2, 1/2, 1\rangle$ state. There are two non-zero Clebsch-Gordan coefficients $C^{F,m}_{F_{A2}, m_{A2}; S, m_S}$ coupling the two A spins:

$$C^{F=3/2, m=1/2}_{F_{A2}=1, m_{A2}=1; S=1/2, m_S=-1/2} = \left\langle 1, 1; \tfrac{1}{2}, -\tfrac{1}{2} \middle| \tfrac{3}{2}, \tfrac{1}{2} \right\rangle = \sqrt{\tfrac{1}{3}},$$

$$C^{F=3/2, m=1/2}_{F_{A2}=1, m_{A2}=0; S=1/2, m_S=1/2} = \left\langle 1, 0; \frac{1}{2}, \frac{1}{2} \middle| \frac{3}{2}, \frac{1}{2} \right\rangle = \sqrt{\frac{2}{3}} \qquad \text{Eq S2}$$

Each of them is multiplied by the corresponding Clebsch-Gordan coefficient coupling the A_2 pair with spin X:

$$C^{F_{A2}=1, m_{A2}=1}_{I_{a1}=1/2, m_{a1}=1/2; I_{a2}=1/2, m_{a2}=1/2} = \left\langle \tfrac{1}{2}, \tfrac{1}{2}; \tfrac{1}{2}, \tfrac{1}{2} \middle| 1,1 \right\rangle = 1,$$

$$C^{F_{A2}=1, m_{A2}=0}_{I_{a1}=1/2, m_{a1}=\pm 1/2; I_{a2}=1/2, m_{a2}=\mp 1/2} = \left\langle \frac{1}{2}, -\frac{1}{2}; \frac{1}{2}, \frac{1}{2} \middle| 1,0 \right\rangle = \left\langle \frac{1}{2}, \frac{1}{2}; \frac{1}{2}, -\frac{1}{2} \middle| 1,0 \right\rangle = \sqrt{\frac{1}{2}} \qquad \text{Eq S3}$$

So that the resulting eigenstate is:

$$|F = 3/2, m = 1/2, F_{A2} = 1\rangle \;=\; \sqrt{\tfrac{1}{3}}\,|\alpha\alpha\beta\rangle + \sqrt{\tfrac{2}{3}}\sqrt{\tfrac{1}{2}}\,(|\alpha\beta\alpha\rangle + |\beta\alpha\alpha\rangle). \qquad \text{Eq S4}$$

Analogously, one can calculate all the eigenstates that are shown in Table S1.1. The reader is encouraged to check this table. Wolfram Mathematica can be used to compute the value of the Clebsch-Gordan coefficients with the syntax ClebschGordan[{Fa1, ma1}, {Fa2, ma2}, {FA2, mA2}].

Finally, we note that the resulting eigenbasis is also the eigenbasis for the A_3 spin system. One may therefore use it to compute the eigenbasis of the *J*-coupling Hamiltonian for the XA_3 spin system using Eq. 46 one more time.

Table S1.1. Eigenstates of XA_2 spin system sorted according to $|F, m, F_{A2}\rangle$ quantum numbers, their explicit forms in terms of Zeeman basis and all the Clebsch-Gordan coefficients that are used to calculate the states. The Clebsch-Gordan coefficients are written using a braket notation with the correspondence $C^{F,m_F}_{I,m_I,S,m_S} = \langle I, S; m_I, m_S|F, m_F\rangle$

**Total spin states** ($\|\boldsymbol{F}, \boldsymbol{m}, \boldsymbol{F_{A2}}\rangle$)	**Explicit form**	**Clebsch-Gordan coefficients used to calculate the eigenstate**
$\|3/2, 3/2, 1\rangle$	$\|\alpha\alpha\alpha\rangle$	**A + A:**<br>$\left\langle \frac{1}{2}, \frac{1}{2}; \frac{1}{2}, \frac{1}{2} \middle\| 1,1 \right\rangle = 1$<br>**$A_2$ + X:**<br>$\left\langle 1, 1; \frac{1}{2}, \frac{1}{2} \middle\| \frac{3}{2}, \frac{3}{2} \right\rangle = 1$
$\|3/2, 1/2, 1\rangle$	$\sqrt{\frac{1}{3}}\|\alpha\alpha\beta\rangle + \sqrt{\frac{2}{3}}\sqrt{\frac{1}{2}}(\|\alpha\beta\alpha\rangle + \|\beta\alpha\alpha\rangle)$	**A + A:**<br>$\left\langle \frac{1}{2}, \frac{1}{2}; \frac{1}{2}, \frac{1}{2} \middle\| 1,1 \right\rangle = 1$<br>$\left\langle \frac{1}{2}, -\frac{1}{2}; \frac{1}{2}, \frac{1}{2} \middle\| 1,0 \right\rangle = \left\langle \frac{1}{2}, \frac{1}{2}; \frac{1}{2}, -\frac{1}{2} \middle\| 1,0 \right\rangle =$<br>$= \sqrt{\frac{1}{2}}$<br>**$A_2$ + X:**<br>$\left\langle 1, 1; \frac{1}{2}, -\frac{1}{2} \middle\| \frac{3}{2}, \frac{1}{2} \right\rangle = \sqrt{\frac{1}{3}}$<br>$\left\langle 1, 0; \frac{1}{2}, \frac{1}{2} \middle\| \frac{3}{2}, \frac{1}{2} \right\rangle = \sqrt{\frac{2}{3}}$
$\|3/2, -1/2, 1\rangle$	$\sqrt{\frac{1}{3}}\|\beta\beta\alpha\rangle + \sqrt{\frac{2}{3}}\sqrt{\frac{1}{2}}(\|\alpha\beta\beta\rangle + \|\beta\alpha\beta\rangle)$	**A + A:**<br>$\left\langle \frac{1}{2}, -\frac{1}{2}; \frac{1}{2}, -\frac{1}{2} \middle\| 1,-1 \right\rangle = 1$<br>$\left\langle \frac{1}{2}, -\frac{1}{2}; \frac{1}{2}, \frac{1}{2} \middle\| 1,0 \right\rangle = \left\langle \frac{1}{2}, \frac{1}{2}; \frac{1}{2}, -\frac{1}{2} \middle\| 1,0 \right\rangle =$<br>$= \sqrt{\frac{1}{2}}$<br>**$A_2$ + X:**<br>$\left\langle 1, -1; \frac{1}{2}, \frac{1}{2} \middle\| \frac{3}{2}, -\frac{1}{2} \right\rangle = \sqrt{\frac{1}{3}}$<br>$\left\langle 1, 0; \frac{1}{2}, -\frac{1}{2} \middle\| \frac{3}{2}, -\frac{1}{2} \right\rangle = \sqrt{\frac{2}{3}}$
$\|3/2, -3/2, 1\rangle$	$\|\beta\beta\beta\rangle$	**A + A:**<br>$\left\langle \frac{1}{2}, -\frac{1}{2}; \frac{1}{2}, -\frac{1}{2} \middle\| 1,-1 \right\rangle = 1$<br>**$A_2$ + X:**<br>$\left\langle 1, -1; \frac{1}{2}, -\frac{1}{2} \middle\| \frac{3}{2}, -\frac{3}{2} \right\rangle = 1$

$\lvert 1/2, 1/2, 1\rangle$	$\sqrt{\frac{2}{3}}\lvert\alpha\alpha\beta\rangle - \sqrt{\frac{1}{3}}\sqrt{\frac{1}{2}}(\lvert\alpha\beta\alpha\rangle + \lvert\beta\alpha\alpha\rangle)$	**A + A:**<br>$\left\langle \frac{1}{2},\frac{1}{2};\frac{1}{2},\frac{1}{2}\middle\vert 1,1\right\rangle = 1$<br>$\left\langle \frac{1}{2},-\frac{1}{2};\frac{1}{2},\frac{1}{2}\middle\vert 1,0\right\rangle = \left\langle \frac{1}{2},\frac{1}{2};\frac{1}{2},-\frac{1}{2}\middle\vert 1,0\right\rangle =$<br>$= \sqrt{\frac{1}{2}}$<br>**$A_2$ + X:**<br>$\left\langle 1,1;\frac{1}{2},-\frac{1}{2}\middle\vert\frac{1}{2},\frac{1}{2}\right\rangle = \sqrt{\frac{2}{3}}$<br>$\left\langle 1,0;\frac{1}{2},\frac{1}{2}\middle\vert\frac{1}{2},\frac{1}{2}\right\rangle = -\sqrt{\frac{1}{3}}$
$\lvert 1/2, -1/2, 1\rangle$	$\sqrt{\frac{2}{3}}\lvert\beta\beta\alpha\rangle - \sqrt{\frac{1}{3}}\sqrt{\frac{1}{2}}(\lvert\alpha\beta\beta\rangle + \lvert\beta\alpha\beta\rangle)$	**A + A:**<br>$\left\langle \frac{1}{2},-\frac{1}{2};\frac{1}{2},-\frac{1}{2}\middle\vert 1,-1\right\rangle = 1$<br>$\left\langle \frac{1}{2},-\frac{1}{2};\frac{1}{2},\frac{1}{2}\middle\vert 1,0\right\rangle == \left\langle \frac{1}{2},\frac{1}{2};\frac{1}{2},-\frac{1}{2}\middle\vert 1,0\right\rangle$<br>$= \sqrt{\frac{1}{2}}$<br>**$A_2$ + X:**<br>$\left\langle 1,-1;\frac{1}{2},-\frac{1}{2}\middle\vert\frac{3}{2},-\frac{1}{2}\right\rangle = \sqrt{\frac{2}{3}}$<br>$\left\langle 1,0;\frac{1}{2},-\frac{1}{2}\middle\vert\frac{3}{2},-\frac{1}{2}\right\rangle = -\sqrt{\frac{1}{3}}$
$\lvert 1/2, 1/2, 0\rangle$	$\sqrt{\frac{1}{2}}(\lvert\alpha\beta\alpha\rangle - \lvert\beta\alpha\alpha\rangle)$	**A + A:**<br>$\left\langle \frac{1}{2},\frac{1}{2};\frac{1}{2},-\frac{1}{2}\middle\vert 0,0\right\rangle = \sqrt{\frac{1}{2}}$<br>$\left\langle \frac{1}{2},-\frac{1}{2};\frac{1}{2},\frac{1}{2}\middle\vert 0,0\right\rangle = -\sqrt{\frac{1}{2}}$<br>**$A_2$ + X:**<br>$\left\langle 0,0;\frac{1}{2},\frac{1}{2}\middle\vert\frac{1}{2},\frac{1}{2}\right\rangle = 1$
$\lvert 1/2, -1/2, 0\rangle$	$\sqrt{\frac{1}{2}}(\lvert\alpha\beta\beta\rangle - \lvert\beta\alpha\beta\rangle)$	**A + A:**<br>$\left\langle \frac{1}{2},\frac{1}{2};\frac{1}{2},-\frac{1}{2}\middle\vert 0,0\right\rangle = -\sqrt{\frac{1}{2}}$<br>$\left\langle \frac{1}{2},-\frac{1}{2};\frac{1}{2},\frac{1}{2}\middle\vert 0,0\right\rangle = -\sqrt{\frac{1}{2}}$<br>**$A_2$ + X:**<br>$\left\langle 0,0;\frac{1}{2},-\frac{1}{2}\middle\vert\frac{1}{2},-\frac{1}{2}\right\rangle = 1$

Supplement 2: Tests on ideal signals to show the efficiency of the Fourier transform algorithm

Supplementary material to "Simulation of NMR spectra at zero- and ultra-low field from A to Z – a tribute to Prof. Konstantin L'vovich Ivanov" https://doi.org/10.5194/mr-2022-18

Q. Stern and K. Sheberstov

This script aims at showing that the Fourier transform is accurately computed. It shows:

- That the frequency axis is accurately computed regardless of the number of points in the FID
- That a window function must be applied to the FID to avoid a baseline shift in the Fourier transform and consequent bias in the signal integration

Effect of zero filling and verification of the frequency axis

This section first computes the time domain signal *St* of an oscillating signal with frequency *nu0* and a decoherence time constant *T2*. The signal is sampled with *N* points from 0 to *tmax*.

```
nu0=10;
T2=6; tmax=30;
N=2^15;
t=linspace(0,tmax,N)'; St=zeros(N,1);
St=St+cos(t*2*pi*nu0).*exp(-t/T2);
```

The signal is Fourier transformed using the function *FFT* (defined at the bottom of the code), with different values of the zero filling *N0*. The real parts of the resulting curves are ploted on the same graph.

The intensity of the signal decreases linearly with the number of points in the Fourier transform (here, *N0*). So that all curves superimpose, each curve is multiplied by the number of points of the Fourier transform *N0*.

```
figure
for i=1:6
    N0=N*i;
    [nu,Snu_1]=FFT(t,St,N0);
    plot(nu,real(Snu_1)*N0,'o-'), hold on
end
hold off
ylabel('Signal intensity (-)')
xlabel('Frequency (Hz)')
x=0.1; xlim(nu0+[-x +x]), grid on
```

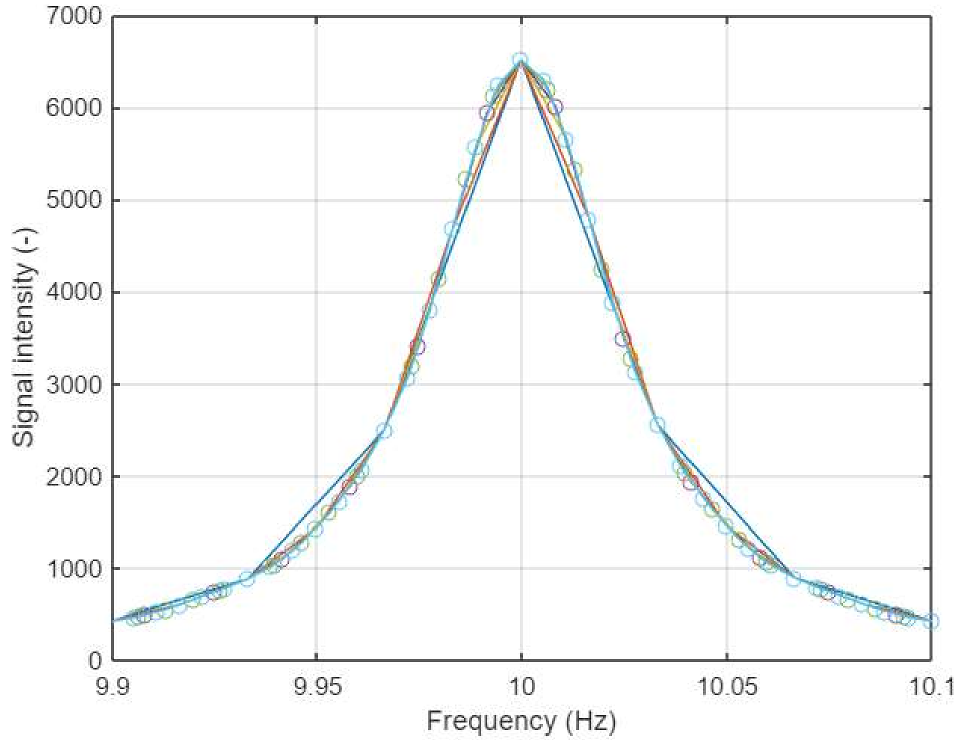

The plot shows that all the curves superimpose perfectly and that the frequency of the signal is precisely *nu0*, as expected. The user may play with the frequency *nu0* and choosing arbitrarily long *T2* values, the signal will always appear at *nu0* (provided *tmax* is chosen to be at least 5**T2*).

This shows that the computation of the Fourier transform and of the frequency axis are precise.

Verification of the consistency of the integration

This section first computes the time domain signal *St* of oscillating signals with a frequency list *nu0* with a decoherence time constant *T2*. The signal is sampled with *N* points from 0 to *tmax*.

```
nu0=[5 25 50];
T2=6; tmax=80;
N=2^15; N0=N*16;
t=linspace(0,tmax,N)'; St=zeros(N,1);
for i=1:length(nu0)
    St=St+cos(t*2*pi*nu0(i)).*exp(-t/T2);
end
```

The signal is Fourier transformed using the functions *FFT* and *FFT_CleanBaseline* (defined at the bottom of the code) with *N0* points.

```
[nu,Snu_1]=FFT_CleanBaseline(t,St,N0);
[nu,Snu_2]=FFT(t,St,N0);
Sreal_1=real(Snu_1);
Sreal_2=real(Snu_2);
```

The real part of the Fourier transform is plotted. The veritical dashed lines show the area of integration of each signal which will be used later, which is defined based on the full width at half maximum of the signal (FWHM) to go from -W*FWHM to +W*FWHM.

```
W=40;
figure
```

```
plot(nu,Sreal_1), hold on
plot(nu,Sreal_2), hold off
grid on
xlim([0 60])
ylabel('Signal intensity (-)'), xlabel('Frequency (Hz)')
for i=1:length(nu0)
    xline(nu0(i)-W/pi/T2,'k--')
    xline(nu0(i)+W/pi/T2,'k--')
end
legend({'FFT using {\itFFTCleanBaseline}','FFT using {\itFFT}'})
```

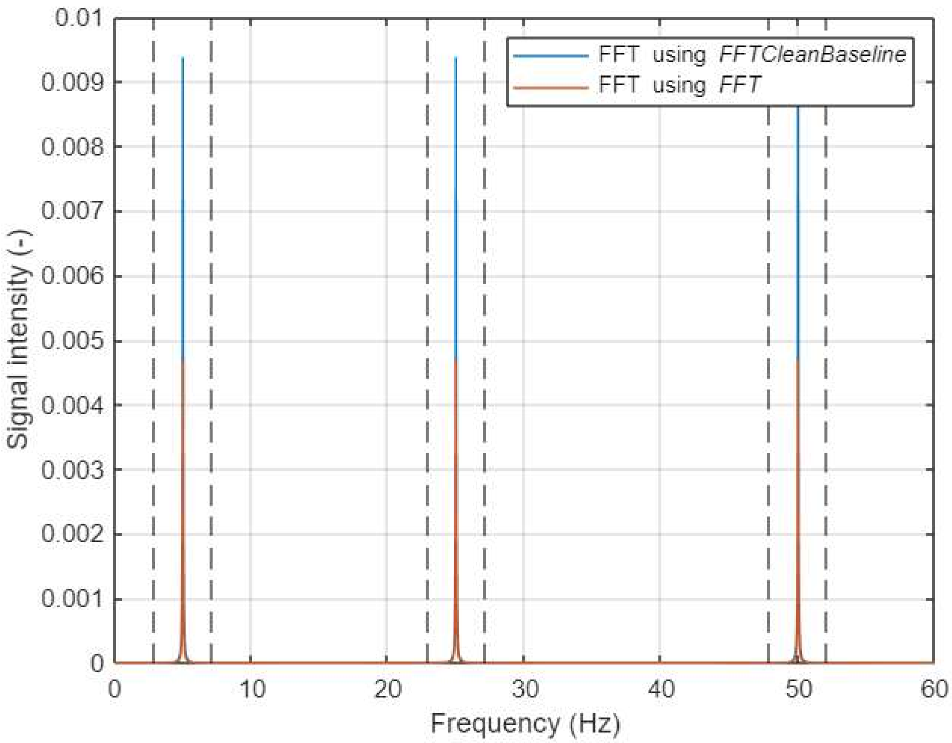

*FFT_CleanBaseline yield*s a signal twice as intense as *FFT*. However, without zooming at the foot of the signals, there is no reason to think that one algorithm is better than the other one.

```
figure
plot(nu,Sreal_1), hold on
plot(nu,Sreal_2), hold off
grid on
xlim([0 60]), ylim([0 1e-5])
ylabel('Signal intensity (-)'), xlabel('Frequency (Hz)')
for i=1:length(nu0)
    xline(nu0(i)-W/pi/T2,'k--')
    xline(nu0(i)+W/pi/T2,'k--')
end
legend({'FFT using {\itFFTCleanBaseline}','FFT using {\itFFT}'})
```

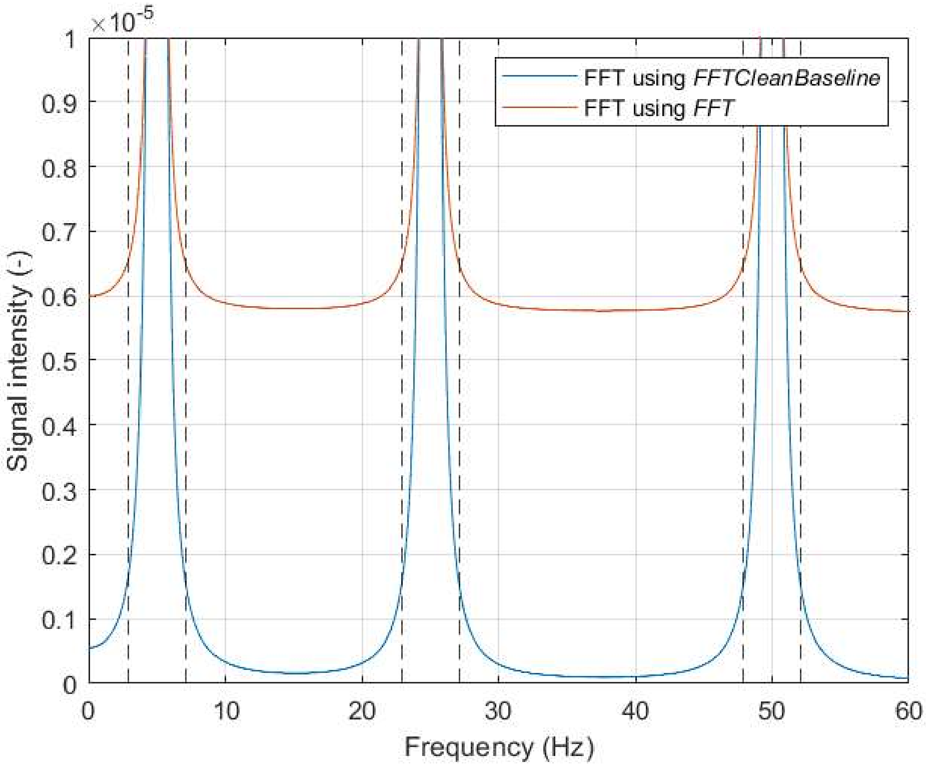

The zoom above reveals that the Fourier transform computed using *FFT* has a baseline shift while that computed using *FFT_CleanBaseline* appropriately tends towards 0 away form the spectral components.

This difference is reflected in the signal integrals that are computed using both Fourier transform algorithms. The intensity of the first point of the FID is, according to Fourier analysis, equal to the total integral of the spectrum. Because the time domain signal is the sum of three cosine waves with unit amplitude, the first point of hte FID is equal to 3:

```
St(1)
```

```
ans = 3
```

For both *FFT* and *FFT_CleanBaseline*, the total sum of the spectrum is equal to the first point of the FID.

In the case of *FFT_CleanBaseline*:

```
sum(Sreal_1)
```

```
ans = 3.0000
```

while in the case of FFT:

```
sum(Sreal_2)
```

```
ans = 3.0000
```

When the individual resonances of the frequency domain signal computed with *FFT_CleanBaseline* are integrated, they tend towards 1, as they should:

```
SumOfTheIntegrals=0;
for i=1:length(nu0)
```

```
    Integral=sum(Sreal_1(nu>nu0(i)-W/pi/T2 & nu<nu0(i)+W/pi/T2));
    disp(['Integral of signal ' num2str(i) ': ' num2str(Integral)])
    SumOfTheIntegrals=SumOfTheIntegrals+Integral;
end
```

```
Integral of signal 1: 0.99258
Integral of signal 2: 0.99226
Integral of signal 3: 0.99214
```

and the sum of the three integrals tends towards 3:

```
SumOfTheIntegrals
```

```
SumOfTheIntegrals = 2.9770
```

To the contrary, when the individual resonances of the frequency domain signal computed with *FFT* are integrated, they tend towards significantly lower values than 1, because part of the integral moved into the baseline:

```
SumOfTheIntegrals=0;
for i=1:length(nu0)
    Integral=sum(Sreal_2(nu>nu0(i)-W/pi/T2 & nu<nu0(i)+W/pi/T2));
    disp(['Integral of signal ' num2str(i) ': ' num2str(Integral)])
    SumOfTheIntegrals=SumOfTheIntegrals+Integral;
end
```

```
Integral of signal 1: 0.52738
Integral of signal 2: 0.52722
Integral of signal 3: 0.52716
```

And the sum of the three integrals is barely higher than half what it should be:

```
SumOfTheIntegrals
```

```
SumOfTheIntegrals = 1.5818
```

```
function [nu,S]=FFT_CleanBaseline(t,I,N)
    % Converts to column vector
    I=I(:);

    % Window function to avoid baseline shift
    I=[I(1); 2*I(2:end)];

    % Sample length (s)
    T = (max(t)-min(t))/(length(I)-1);

    % Frequency axis (Hz)
    nu = (0:N/2-1)/T/N;

    % Fourier transform
    S = fft(I,N)/N;

    % Remove negative frequencies
    S=2*S(1:N/2);
end
function [nu,S]=FFT(t,I,N)
    % Converts to column vector
    I=I(:);
```

```
    % Sample length (s)
    T = (max(t)-min(t))/(length(I)-1);

    % Frequency axis (Hz)
    nu = (0:N/2-1)/T/N;

    % Fourier transform
    S = fft(I,N)/N;

    % Remove negative frequencies
    S=2*S(1:N/2);
end
```

Supplement 3: Simulation of NMR spectra at zero and ultra-low field vs high field

Supplementary material to "Simulation of NMR spectra at zero- and ultra-low field from A to Z – a tribute to Prof. Konstantin L'vovich Ivanov" https://doi.org/10.5194/mr-2022-18

Q. Stern and K. Sheberstov

This script shows how to simulate NMR spectra for a pair of *J*-coupled spins (1H and 13C in this example) at zero field (ZF), ultra-low field (ULF) and high field (HF). The code follows pricesely the structure of the paper.

```
clear all
```

All simulated experiments are performed at 298 K (use to compute the thermal equilibrium polarizations)

```
T=298;              % Temperature of prepolarization in K
```

Physical constants (used to compute the thermal equilibrium polarizations and the magnetic field produced by the sample in the ZULF experiments)

```
hbar=1.05457e-34;   % Reduced Planck constant in J.s/rad
kB=1.3806485e-23;   % Boltzmann constant J.K-1
mu0=4*pi*1e-7;      % Permeability of free space dived in T.m.A-1
NA=6.02e23;         % Avogadro's number in mol-1
```

2.1 Define the experimental sequence

ZF case

The sample is prepolarized at 2 T (magnetic field aligned along the *z*-axis). We assume that the sample spends enough time in the prepolarizing field for the spins to be at equilibrium (Boltzmann distribution). The sample is brought suddenly to zero field, where the magnetic field generated by the sample along time is measured by a magnetometer placed along the *z*-axis and sensitive to fields along the *z*-axis. The sudden transitions projects the Zeeman states onto the basis states at ZF (the states of the coupled basis) and induce coherences between them.

```
Bpol=2;             % Field for prepolarization in T
```

ULF case

The experiment is the same as the ZF, except that a magnetic field of 50 uT is applied along the *x*-axis during detection.

```
Bx=0.5e-6;          % Magnetic field along x for ULF experiments in T
```

HF case

Standard HF-NMR acquisition at 9.4 T where a 90° radiofrequency (rf) pulse along the *x*-axis is applied to nutate the spins from the *z*-axis to the *y*-axis. The oscillating magnetic field is recorded along the *y*-axis. The experiment is simulated both for pulse and acquisition on 1H and 13C.

```
B0=9.4;             % Magnetic field for HF experiments in T (typical 400 MHz magnet)
```

2.2 Define the spin system

The spin system is a pair of *J*-coupled spins. Spins I and S are a 13C and 1H spin, respectively, for all simulations.

```
JIS=140;            % J-coupling between spin I and S in Hz
gI= 67.262e6;       % Gyromagnetic ration of spin I (13C) in rad.s-1.T-1
gS=276.513e6;       % Gyromagnetic ration of spin S (1H) in rad.s-1.T-1
```

2.3 Compute the spin Hamiltonian

Both the ZULF and HF simulation use the same operators. Their expression is given in Sec. 2.3 of the manuscript

Pauli matrices (see Eq. 3)

```
e =[1 0;  0 1];
sx=[0 1;  1 0];
sy=[0 -1i;1i 0];
sz=[1 0;  0 -1];
```

Operators for spin I, i.e., the 13C spin (see Eq. 6)

```
Ix=kron(sx/2,e);
Iy=kron(sy/2,e);
Iz=kron(sz/2,e);
```

Operators for spin S, i.e., the 1H spin (see Eq. 7)

```
Sx=kron(e,sx/2);
Sy=kron(e,sy/2);
Sz=kron(e,sz/2);
```

Unity operator in the two-spin Hilbert space

```
E=kron(e,e);
```

Zeeman Hamiltonian expressed in rad.s-1 as a function of field along x, y and z in T

```
HZ=@(Bx, By, Bz) -gI*(Bx*Ix+By*Iy+Bz*Iz) -gS*(Bx*Sx+By*Sy+Bz*Sz);
```

J-Hamiltonian expressed in rad.s-1 (with the *J*-coupling expressed in Hz)

```
HJ=2*pi*JIS*(Ix*Sx+Iy*Sy+Iz*Sz);
```

Total Hamiltonian expressed in rad.s-1 as a function of field along x, y and z in T

```
Htot=@(Bx, By, Bz) HJ+HZ(Bx, By, Bz);
```

2.4 Define the initial state: compute the initial density matrix

ZULF case

Polarization of spin I and S at equilibrium in the prepolarizing magnet calculated using Boltzman distribution (see Eq. 22)

```
PI=tanh(hbar*Bpol*gI/kB/T/2);
PS=tanh(hbar*Bpol*gS/kB/T/2);
```

The initial state is the same for the ZF and ULF experiment (see Eq. 23)

```
Iz_2x2=sz/2;
rhoI=e/2+PI*Iz_2x2;
rhoS=e/2+PS*Iz_2x2;
rho_eq_ZULF=kron(rhoI,rhoS);
```

Modification of the density matrix removing the identity (see Eq. 24)

```
rho_eq_ZULF=rho_eq_ZULF-E/4;
```

HF case

The only diffrence with the ZULF case is that the magnetic field at which the spins are prepolarized is higher at HF.

Polarization of spin I and S at equilibrium in the 9.4 T NMR magnet calculated using Boltzman distribution (see Eq. 22)

```
PI=tanh(hbar*B0*gI/kB/T/2);
PS=tanh(hbar*B0*gS/kB/T/2);
```

The initial state is the same for the ZF and ULF experiment (see Eq. 23)

```
Iz_2x2=sz/2;
rhoI=e/2+PI*Iz_2x2;
```

```
rhoS=e/2+PS*Iz_2x2;
rho_eq_HF=kron(rhoI,rhoS);
```

Modification of the density matrices removing the identity (see Eq. 24)

```
rho_eq_HF=rho_eq_HF-E/4;
```

2.5-6 Propagate the density matrix under the Hamiltonians and extract expectation values from the propagation

In practice the expectation values are extracted from the density matrix during the progation (and not in an extra step). This avoids creating a second *for* loop.

Parameters for propagation

```
taq=5;              % Propagation/acquisition time in s
K=2^12;             % Number of points in the FID
L=2^16;             % Number of points in the FID with zero filling
T2=1;               % Coherence time constant in s
dt=taq/K;           % Time interval for propagation in s
t=(0:K-1)'/K*taq;   % Time axis in s
```

ZF case

Observable operator: total field along z in pT at distance r from the sample (in the point dipole approximation, see Eq. 34 and 35). The sample contains 27 mol-L-1 of molecules and has a volume of 100 uL. The detector is placed at 1 cm from the sample.

```
C=27;               % Molecule concentration in mol.L-1
V=100e-6;           % Sample volume in L
r=0.01;             % Distance between the center of the sample and the center of the detector in m
N=NA*C*V;           % Number of molecules
O=gI*Iz+gS*Sz;      % Observation operator
Constant=N*hbar*1e12*mu0/2/pi/r^3;
```

Propagator during time interval dt with no field along any direction (see Eq. 30)

```
U =expm(-1i*dt*Htot(0,0,0));
```

Empty vector to store the time domain signal during the for loop

```
TDS_ZF=zeros(K,1);
```

Initialization of the density matrix

```
rho=rho_eq_ZULF;
```

Propagation loop

Propagation loop. The first line in the loop computes the expectation value of the field along z (see Eq. 35) at time t_k. The second line in the loop propagates the state of the system during the interval dt, in other words, it "takes" the density matrix from time t_k to t_{k+1} (see Eq. 29). Note that each loop overrides the previous density matrix. The expectation value that is computed at iteration *k* corresponds to the density matrix of iteration *k*-1*.* Note that the expectation value should in principle be real-valued. However, due to the finite precision of numerical calculations, some minute imaginary part may remain when computing the trace. We therefore use the function *real()* to remove the imaginary part.

```
for k=1:K
    TDS_ZF(k)=Constant*real(trace(O*rho));
    rho=U*rho*U';
end
```

ULF case

The observable is the same as for the ZF case.

Propagator during time interval dt with a bias field along the *x*-axis and no fields along the *y*- and *z*-axes (see Eq. 30).

```
U=expm(-1i*dt*Htot(Bx,0,0));
```

Empty vector to store the time domain signal during the for loop

```
TDS_ULF=zeros(K,1);
```

Initialization of the density matrix

```
rho=rho_eq_ZULF;
```

Propagation loop. The first line in the loop computes the expectation value of the field along z (see Eq. 35) at time t_k. The process is exaclty the same as for the ZF case, except that the propagator was calculated for the ULF Hamiltonian (with a small bias field along the *x*-axis).

```
for k=1:K
    TDS_ULF(k)=Constant*real(trace(O*rho));
    rho=U*rho*U';
end
```

HF case - 13C

The observable at high field is the a operator (expressed in arbitrary units)

```
O=Ix-1i*Iy;
```

Propagator with a bias field along the *x*-axis and no fields along the *y*- and *z*-axes (see Eq. 30).

```
U=expm(-1i*dt*Htot(0,0,B0));
```

Empty vector to store the time domain signal during the for loop

```
TDS_HF_13C=zeros(K,1);
```

Initialization of the density matrix

```
rho=rho_eq_HF;
```

The HF experiment requires simulating a pulse: the thermal equilibrium density matrix represents both the 13C and 1H spins with polarization along the *z*-axis. The rotation operator *Up* below (see the discussion following Eq. 29) is used to rotate the 13C spin from the *z*-axis to the the *y*-axis.

```
Up=expm(-1i*Iy*pi/2);
rho=Up*rho*Up';
```

Propagation loop. The first line in the loop computes the expectation value of the field along z (see Eq. 35) at time t_k. The second line in the loop propagates the state of the system during the interval dt, in other words, it "takes" the density matrix from time t_k to t_{k+1} (see Eq. 29).

```
for k=1:K
    TDS_HF_13C(k)=trace(O*rho);
    rho=U*rho*U';
end
```

HF case - 1H

The observable at high field is a shift operator (expressed in arbitrary units)

```
O=Sx-1i*Sy;
```

Propagator with a bias field along the *x*-axis and no fields along the *y*- and *z*-axes (see Eq. 30).

```
U=expm(-1i*dt*Htot(0,0,B0));
```

Empty vector to store the time domain signal during the for loop

```
TDS_HF_1H=zeros(K,1);
```

Initialization of the density matrix

```
rho=rho_eq_HF;
```

The HF experiment requires simulating a pulse: the thermal equilibrium density matrix represents both the 13C and 1H spins with polarization along the *z*-axis. The rotation operator *Up* below (see the discussion following Eq. 29) is used to rotate the 1H spin from the *z*-axis to the the *y*-axis.

```
Up=expm(-1i*Sy*pi/2);
rho=Up*rho*Up';
```

Propagation loop. The first line in the loop computes the expectation value of the field along z (see Eq. 35) at time t_k. The second line in the loop propagates the state of the system during the interval dt, in other words, it "takes" the density matrix from time t_k to t_{k+1} (see Eq. 29).

```
for k=1:K
    TDS_HF_1H(k)=trace(O*rho);
    rho=U*rho*U';
end
```

2.7 Fourier transform the expectation values to obtain a spectrum

The time domain signals obained in the previous step do not decay to zero because we did not include any relaxation in the simulations, as can be seen in the plots below.

```
figure
subplot(2,2,1)
plot(t,TDS_ZF)
ylabel('<{\itB}_z> (pT)')
xlim([0 max(t)])
title('ZF')

subplot(2,2,2)
plot(t,TDS_ULF)
xlim([0 max(t)])
title('ULF')

subplot(2,2,3)
plot(t,real(TDS_HF_13C))
xlabel('Time (s)'), ylabel('Real signal (a. u.)')
xlim([0 max(t)])
title('HF - ^{13}C')

subplot(2,2,4)
plot(t,real(TDS_HF_1H))
xlabel('Time (s)')
xlim([0 max(t)])
title('HF - ^1H')
```

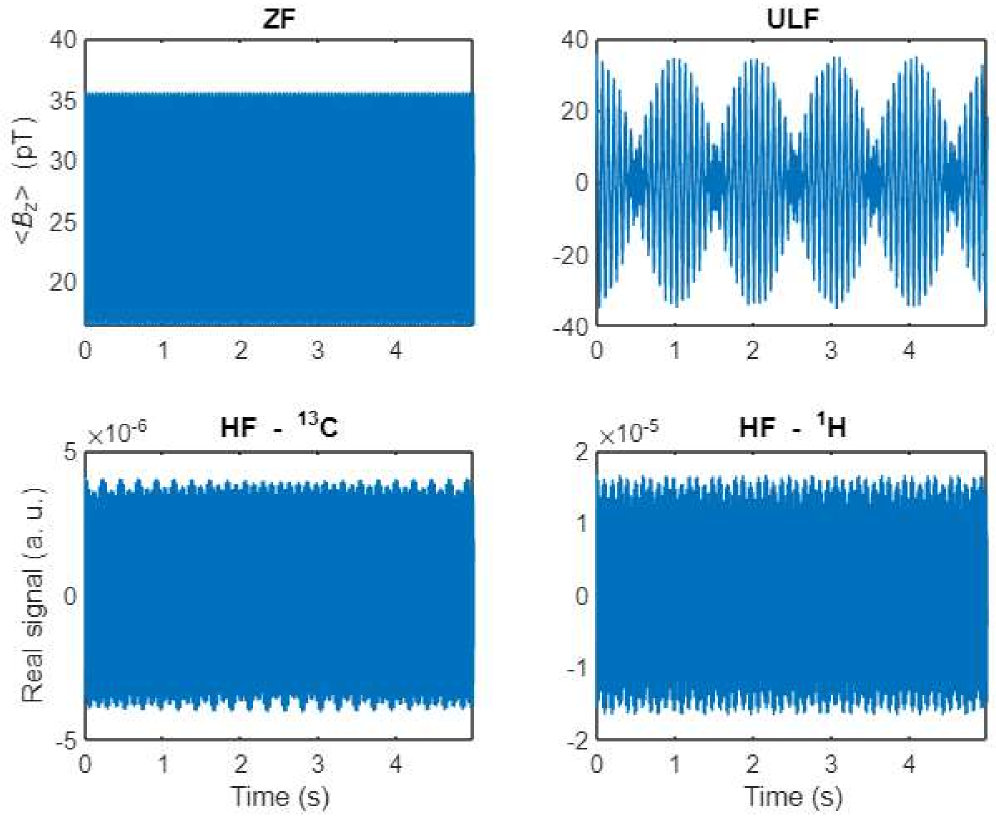

To make the signals decay 0, we apply an apodization function (see Eq. 37)

```
TDS_ZF2     =TDS_ZF     .*exp(-t/T2);
TDS_ULF2    =TDS_ULF    .*exp(-t/T2);
TDS_HF_13C2=TDS_HF_13C.*exp(-t/T2);
TDS_HF_1H2 =TDS_HF_1H .*exp(-t/T2);

figure
subplot(2,2,1)
plot(t,TDS_ZF2)
ylabel('<{\itB}_z> (pT)')
xlim([0 max(t)])
title('ZF')

subplot(2,2,2)
plot(t,TDS_ULF2)
xlim([0 max(t)])
title('ULF')

subplot(2,2,3)
plot(t,real(TDS_HF_13C2))
xlabel('Time (s)'), ylabel('Real signal (a. u.)')
xlim([0 max(t)])
title('HF - ^{13}C')

subplot(2,2,4)
plot(t,real(TDS_HF_1H2))
xlabel('Time (s)')
xlim([0 max(t)])
title('HF - ^1H')
```

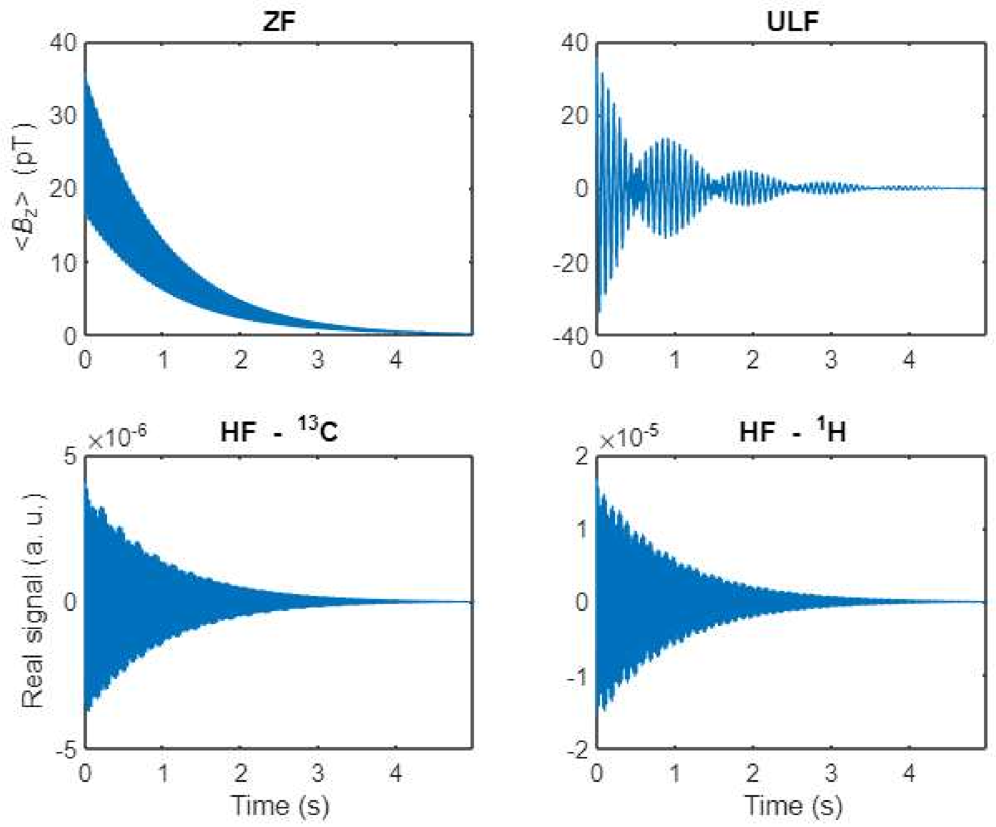

A second apodization function is applied to avoid baseline distortion (see Eq. 37 and the related discussion)

```
TDS_ZF3     =[TDS_ZF2(1);      2*TDS_ZF2(2:end-1);     TDS_ZF2(end)];
TDS_ULF3    =[TDS_ULF2(1);     2*TDS_ULF2(2:end-1);    TDS_ULF2(end)];
TDS_HF_13C3=[TDS_HF_13C2(1); 2*TDS_HF_13C2(2:end-1);TDS_HF_13C2(end)];
TDS_HF_1H3 =[TDS_HF_1H2(1);  2*TDS_HF_1H2(2:end-1); TDS_HF_1H2(end)];
```

At HF, the time-domain signal must also be modulated by multiplying the time-domain signal by a complex exponential. This mathematical process is equivalent to what the spectrometer does: the NMR coil picks up a signal at oscillate at the Larmor frequency, which is much too high to be digitized. The oscillating current is therefore modulated with the carrier frequency and the modulated signal is digitized. This shifts the frequency of the oscillating signals and cause the spectrum to be centered around the carrier frequency.

```
omega1=-gI*B0;
omega2=-gS*B0;
TDS_HF_13C4=TDS_HF_13C3.*exp(1i*omega1*t);
TDS_HF_1H4 =TDS_HF_1H3 .*exp(1i*omega2*t);

figure('Units','centimeters','Position',[0 0 14.8167   11.1125/2])
subplot(1,2,1)
plot(t,real(TDS_HF_13C4))
xlabel('Time (s)'), ylabel('Real signal (a. u.)')
xlim([0 max(t)])
title('HF - ^{13}C')

subplot(1,2,2)
plot(t,real(TDS_HF_1H4))
xlabel('Time (s)')
xlim([0 max(t)])
title('HF - ^1H')
```

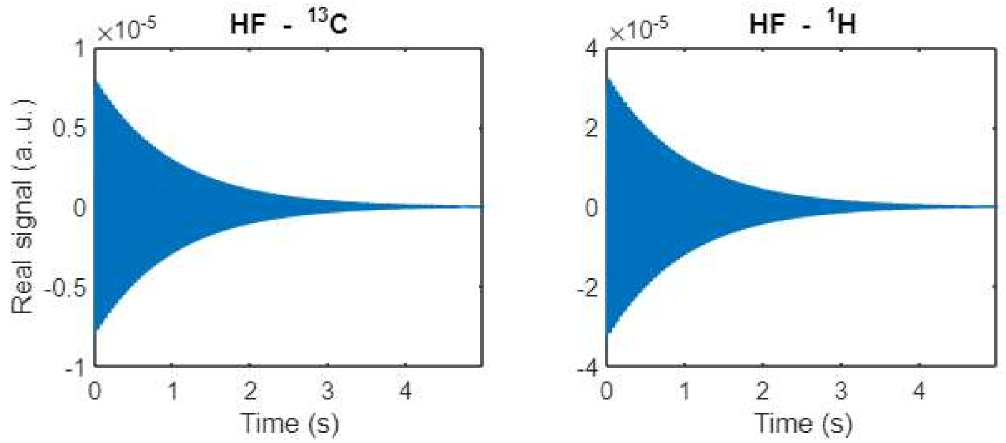

Finally, the signal is Fourier transformed to obtain the frequency-domain signal. In the case of the ZULF signals, only the real part of the Fourier transform is retained.

```
FDS_ZF = real(fft(TDS_ZF3,L))/L;
FDS_ULF= real(fft(TDS_ULF3,L))/L;
FDS_ZF = FDS_ZF(1:L/2);
FDS_ULF= FDS_ULF(1:L/2);
```

At HF, the measured signal is complex. Note that the functions *fftshift()* is used for the signals at HF because the convention that MATLAB uses for the fast Fourier transform does not places the 0 frequency on the edges. To obtain a frequency-domain as is common in NMR, the function *fftshift()* is used.

```
FDS_HF_13C = fftshift(fft(TDS_HF_13C4,L))/L;
FDS_HF_1H  = fftshift(fft(TDS_HF_1H4,L))/L;
```

The frequency axis is generated using Eq. 39 and 40. At ZULF, the time-domain signal is real (in usual cases) and so only positive frequencies are used. At HF, the time-domain signal is imaginary and so both negative and positive frequencies are used.

```
f=(K-1)/taq;             % Sampling frequency in Hz
nu_ZULF=(0:L/2-1)/L*f;  % Frequency axis in Hz
nu_HF=(-L/2:L/2-1)/L*f; % Frequency axis in Hz
```

The figure below plots the four obtained spectra. The ZF spectrum contains one line at 0 and one at the frequency of the *J*-coupling. These lines are split at ULF. Both spectra at HF have the same appearance: they consist of doublet centered on the carrier frequency (because our simulation did not include any chemical shift) with the splitting of the doublet corresponding to the frequency of the *J*-coupling. The signals on the HF 1H spectrum is 4x more intense than that of the 13C HF spectrum because of the 4x higher Boltzmann polarization of 1H spins.

```
figure
subplot(2,2,1)
plot(nu_ZULF,FDS_ZF)
xlabel('Frequency (Hz)')
ylabel('<{\itB}_z> (pT)')
xlim([0 180])
title('ZF')

subplot(2,2,2)
plot(nu_ZULF,FDS_ULF)
xlabel('Frequency (Hz)')
xlim([0 180])
title('ULF')

subplot(2,2,3)
plot(nu_HF,real(FDS_HF_13C))
xlabel('^{13}C Frequency (Hz)'), ylabel('Real signal (a. u.)')
xlim([-100 100])
title('HF - ^{13}C')
```

```
subplot(2,2,4)
plot(nu_HF,real(FDS_HF_1H))
xlabel('^1H Frequency (Hz)')
xlim([-100 100])
title('HF - ^1H')
```

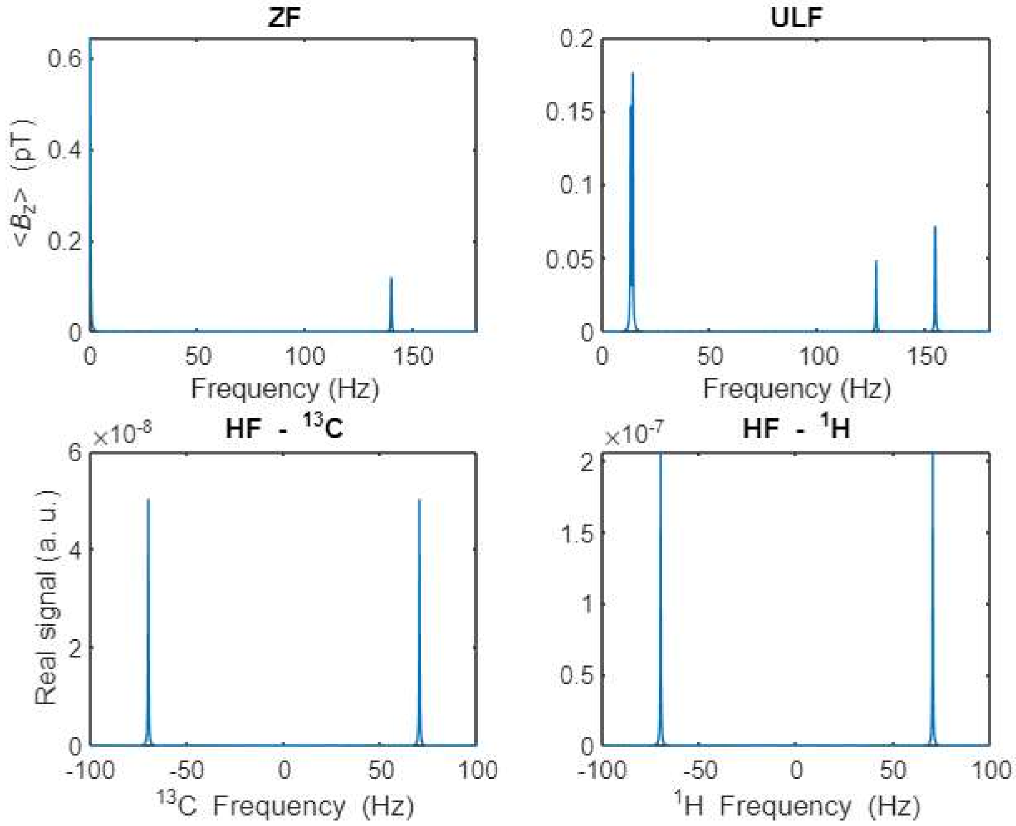

Supplement 4: Simulation of NMR spectra at zero and ultra-low field - Case of XA spin system

Supplementary material to "Simulation of NMR spectra at zero- and ultra-low field from A to Z – a tribute to Prof. Konstantin L'vovich Ivanov" https://doi.org/10.5194/mr-2022-18

Q. Stern and K. Sheberstov

This script shows how to simulate ZULF spectra for an XA spin system. It was used to generate Figures 4 and 5 of the paper.

Figure path and plot options

```
clear all
FigurePath='C:\Users\quent\Documents\Projects\Tutorial paper on ZULF\Figures\';
FigureWidth=11;             % Figure width in cm
FigureHeight=3;             % Figure height in cm
FigureWidth2=12;            % Nutation figure width in cm
FigureHeight2=3;            % Nutation figure height in cm
c1=[0    0.4470    0.7410]; % Figure color 1
c2=[1 1 1]*0.5;             % Figure color 2
lw=1;                       % Figure linewidth
YlimSpectra=[0 0.4];        % Figure spectra y-limits in pT
YlimFID=[-60 60];           % Figure FID y-limits in pT
Xlim=[0 160];               % Figure spectra x-limits
```

General paramaters and operators

Spin system paramaters

```
JIS=140;            % J-coupling between I and S in Hz
gI= 67.262e6;       % Gyromagnetic ration of spin I (13C) in rad.s-1.T-1
gS=276.513e6;       % Gyromagnetic ration of spin S (1H) in rad.s-1.T-1
```

Experimental conditions

```
Bpol=2;             % Field for prepolarization in T
T=298;              % Temperature of prepolarization in K
Bx=0.5e-6;          % Magnetic field along x for ultra-low field experiments in T
Bpulse=50e-6;       % Magnetic field along x during DC pulses in T
NA=6.02e23;         % Avogadro's number in mol-1
C=27;               % Molecule concentration in mol.L-1
V=100e-6;           % Sample volume in L
r=0.01;             % Distance between the center of the sample and the center of the detector in m
```

Physical constants

```
hbar=1.05457e-34;   % Reduced Planck constant in J.s/rad
kB=1.3806485e-23;   % Boltzmann constant J.K-1
mu0=4*pi*1e-7;      % Permeability of free space dived in T.m.A-1
```

Parameters for propagation

```
taq=5;              % Propagation/acquisition time in s
K=2^12;             % Number of points in the FID
L=2^16;             % Number of points in the FID with zero filling
T2=1;               % Coherence time constant in s
dt=taq/K;           % Time interval for propagation in s
t=(0:K-1)'/K*taq;   % Time axis in s
```

Frequency axis

```
f=(K-1)/taq;        % Sampling frequency in Hz
nu=(0:L/2-1)/L*f;   % Frequency axis in Hz (only positive frequencies)
```

Integration range

```
numin=138;          % Lower boundary for signal integration in Hz
numax=142;          % Higher boundary for signal integration in Hz
```

Definition of operators

Pauli matrices

```
e =[1 0;  0 1];
sx=[0 1;  1 0];
sy=[0 -1i;1i 0];
sz=[1 0;  0 -1];
```

Operators for spin I

```
Ix=kron(sx/2,e);
Iy=kron(sy/2,e);
Iz=kron(sz/2,e);
```

Operators for spin S

```
Sx=kron(e,sx/2);
Sy=kron(e,sy/2);
Sz=kron(e,sz/2);
```

Unity operator

```
E=kron(e,e);
```

Initial density matrix

Polarization of spin I at equilibrium in the prepolarizing magnet calculated using Boltzman distribution

```
PI=tanh(hbar*Bpol*gI/kB/T/2);
```

Polarization of spin S at equilibrium in the prepolarizing magnet calculated using Boltzman distribution

```
PS=tanh(hbar*Bpol*gS/kB/T/2);
```

Initial density matrix of spin I and S at equilibrium in the prepolarizing magnet

```
Iz_2by2=sz/2;
rhoI=e/2+PI*Iz_2by2;
rhoS=e/2+PS*Iz_2by2;
rho_eq=kron(rhoI,rhoS);
```

Modification of the density matrix removing the identity

```
NumberOfSpins=2;
rho_eq=rho_eq-eye(2^NumberOfSpins)/2^NumberOfSpins;
```

Hamiltonian and propagators

Zeeman Hamiltonian as a function of field along x, y and z

```
HZ=@(Bx, By, Bz) -gI*(Bx*Ix+By*Iy+Bz*Iz) -gS*(Bx*Sx+By*Sy+Bz*Sz);
```

J Hamiltonian

```
HJ=2*pi*JIS*(Ix*Sx+Iy*Sy+Iz*Sz);
```

Total Hamiltonian

```
H=@(Bx, By, Bz) HJ+HZ(Bx, By, Bz);
```

Propagators at zero and ultra-low field

```
U_ZF =expm(-1i*dt*H(0,0,0));
U_ULF=expm(-1i*dt*H(Bx,0,0));
```

Observable operator: total field along z in pT at distance r from the sample (in the point dipole approximation)

```
N=NA*C*V;
O=(gI*Iz+gS*Sz)*N*hbar*1e12*mu0/2/pi/r^3;
```

Sudden drop experiments

Zero field case

```
% Vector for the time domain signal
TDS_sudden_ZF=zeros(K,1);

% Initialization of the density matrix
rho=rho_eq;
for k=1:K
    % Computes the expectation value of the field along z
    TDS_sudden_ZF(k)=real(trace(O*rho));

    % Propagates during dt
    rho=U_ZF*rho*U_ZF';
end

% Apodization - line broadening
TDS_sudden_ZF=TDS_sudden_ZF.*exp(-t/T2);

% Fourier transform
FDS_sudden_ZF = FourierTransform(TDS_sudden_ZF,L);

% Intensity of the sudden drop signal
I_sudden_drop=sum(FDS_sudden_ZF(nu>numin & nu<numax))
```

```
I_sudden_drop = 9.1326
```

Ultra-low field case

```
% Vector for the time domain signal
TDS_sudden_ULF=zeros(K,1);

% Initialization of the density matrix
rho=rho_eq;
for k=1:K
    % Computes the expectation value of the field along z
    TDS_sudden_ULF(k)=real(trace(O*rho));

    % Propagates during dt
    rho=U_ULF*rho*U_ULF';
end

% Relaxation
TDS_sudden_ULF=TDS_sudden_ULF.*exp(-t/T2);

% Fourier transform
FDS_sudden_ULF = FourierTransform(TDS_sudden_ULF,L);
```

Plot the results

```
figure("Units","centimeters","Position",[0 0 FigureWidth FigureHeight])
subplot(1,4,1)
plot(t,TDS_sudden_ULF,'LineWidth',lw,'color',c2), hold on
plot(t,TDS_sudden_ZF,'LineWidth',lw,'color',c1), hold off
xlim([0 taq]), %xlabel('Time (s)')
ylabel('<{\itB}_z> (pT)')
ylim(YlimFID)
set(gca,'fontsize',7)

subplot(1,4,[2 4])
plot(nu,FDS_sudden_ULF,'LineWidth',lw,'color',c2), hold on
plot(nu,FDS_sudden_ZF,'LineWidth',lw,'color',c1), hold off
xlim(Xlim), %xlabel('Frequency (Hz)')
set(gca,'fontsize',7)
```

```
ylim(YlimSpectra)

exportgraphics(gcf,fullfile(FigurePath, 'XA_system_sudden_drop.pdf'),'ContentType','vector')
```

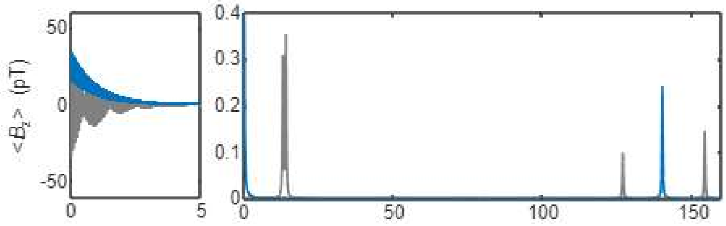

Adiabatic field drop

Propagation of the adiabatic field drop

```
B_start=200e-6; % Magnetic field at the start of the decay in T
t_adia=0.5;     % Time of the adiabatic field drop in s
T_adia=0.05;    % Decay time constant of the adiabatic field drop in s
M=5000;         % Number of discrete points for the adiabatic field drop
```

Time intervals for adiabatic field drop

```
dt_adia=t_adia/M;
```

Field profile of the adiabatic field drop: exponential decay generated by a Helmholtz coil.

```
B_adia=B_start*(exp(-(0:M-1)/M*t_adia/T_adia)-exp(-t_adia/T_adia))/(1-exp(-t_adia/T_adia));
```

Propagation

```
rho_adia=rho_eq;
for k=1:M
    U=expm(-1i*dt_adia*H(0,0,B_adia(k)));
    rho_adia=U*rho_adia*U';
end
```

Zero field case

```
% Vector for the time domain signal
TDS_AFD_ZF=zeros(K,1);

% Initialization of the density mat
rho=rho_adia;
for k=1:K
    % Computes the expectation value of the field along z
    TDS_AFD_ZF(k)=real(trace(O*rho));

    % Propagates during dt
    rho=U_ZF*rho*U_ZF';
end

% Apodization - line broadening
TDS_AFD_ZF=TDS_AFD_ZF.*exp(-t/T2);

% Fourier transform
FDS_AFD_ZF = FourierTransform(TDS_AFD_ZF,L);
```

Ultra-low field case

Propagation

```
% Vector for the time domain signal
TDS_AFD_ULF=zeros(K,1);

% Initialization of the density mat
rho=rho_adia;
for k=1:K
```

```
    % Computes the expectation value of the field along z
    TDS_AFD_ULF(k)=real(trace(O*rho));

    % Propagates during dt
    rho=U_ULF*rho*U_ULF';
end

% Relaxation
TDS_AFD_ULF=TDS_AFD_ULF.*exp(-t/T2);

% Fourier transform
FDS_AFD_ULF = FourierTransform(TDS_AFD_ULF,L);
```

Plot the results

```
figure("Units","centimeters","Position",[0 0 FigureWidth FigureHeight])
subplot(1,4,1)
plot(t,TDS_AFD_ULF,'LineWidth',lw,'color',c2), hold on
plot(t,TDS_AFD_ZF,'LineWidth',lw,'color',c1), hold off
xlim([0 taq]), %xlabel('Time (s)')
ylim(YlimFID), ylabel('<{\itB}_z> (pT)')
set(gca,'fontsize',7)

subplot(1,4,[2 4])
plot(nu,FDS_AFD_ULF,'LineWidth',lw,'color',c2), hold on
plot(nu,FDS_AFD_ZF,'LineWidth',lw,'color',c1), hold off
xlim(Xlim), %xlabel('Frequency (Hz)')
set(gca,'fontsize',7)
ylim(YlimSpectra)

exportgraphics(gcf,fullfile(FigurePath, 'XA_system_adiabatic.pdf'),'ContentType','vector')
```

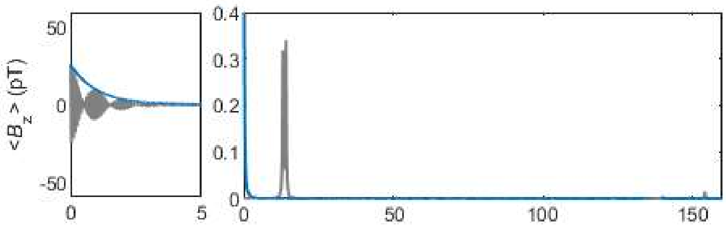

Propgation of the pulse along Z

Pulse length in s

```
tpulse=150e-6;
```

Pulse propagator and density operator after the pulse

```
Upulse=expm(-1i*tpulse*HZ(0,0,Bpulse));
rho_afterpuse=Upulse*rho_adia*Upulse';
```

Zero field case

```
% Vector for the time domain signal
TDS_Z_pulse_ZF=zeros(K,1);

% Initialization of the density mat
rho=rho_afterpuse;
for k=1:K
    % Computes the expectation value of the field along z
    TDS_Z_pulse_ZF(k)=real(trace(O*rho));

    % Propagates during dt
    rho=U_ZF*rho*U_ZF';
end

% Apodization - line broadening
TDS_Z_pulse_ZF=TDS_Z_pulse_ZF.*exp(-t/T2);
```

```
% Fourier transform
phase=pi/2;
FDS_Z_pulse_ZF = FourierTransform(TDS_Z_pulse_ZF*exp(1i*phase),L);
```

Ultra-low field case

```
% Vector for the time domain signal
TDS_Z_pulse_ULF=zeros(K,1);

% Initialization of the density mat
rho=rho_afterpuse;
for k=1:K
    % Computes the expectation value of the field along z
    TDS_Z_pulse_ULF(k)=real(trace(O*rho));

    % Propagates during dt
    rho=U_ULF*rho*U_ULF';
end

% Relaxation
TDS_Z_pulse_ULF=TDS_Z_pulse_ULF.*exp(-t/T2);

% Fourier transform
phase=pi/2;
FDS_Z_pulse_ULF = FourierTransform(TDS_Z_pulse_ULF*exp(1i*phase),L);
```

Plot the results

```
figure("Units","centimeters","Position",[0 0 FigureWidth FigureHeight])
subplot(1,4,1)
plot(t,TDS_Z_pulse_ULF,'LineWidth',lw,'Color',c2), hold on
plot(t,TDS_Z_pulse_ZF,'LineWidth',lw,'Color',c1), hold off
xlim([0 taq]), %xlabel('Time (s)')
ylim(YlimFID), ylabel('<{\itB}_z> (pT)')
set(gca,'fontsize',7)

subplot(1,4,[2 4])
plot(nu,FDS_Z_pulse_ULF,'LineWidth',lw,'Color',c2), hold on
plot(nu,FDS_Z_pulse_ZF,'LineWidth',lw,'Color',c1), hold off
xlim(Xlim), %xlabel('Frequency (Hz)')
set(gca,'fontsize',7)
ylim(YlimSpectra)

exportgraphics(gcf,fullfile(FigurePath, 'XA_system_adiabatic_and_pulse_Z.pdf'),'ContentType','vector')
```

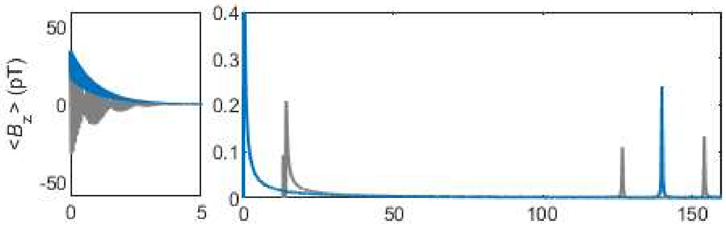

Pulse along X

Pulse length in s

```
tpulse=910e-6;
```

Pulse propagator and density operator after the pulse

```
Upulse=expm(-1i*tpulse*HZ(Bpulse,0,0));
rho_afterpuse=Upulse*rho_adia*Upulse';
```

Zero field case

```
% Vector for the time domain signal
```

```
TDS_X_pulse_ZF=zeros(K,1);

% Initialization of the density mat
rho=rho_afterpuse;
for k=1:K
    % Computes the expectation value of the field along z
    TDS_X_pulse_ZF(k)=real(trace(O*rho));

    % Propagates during dt
    rho=U_ZF*rho*U_ZF';
end

% Apodization - line broadening
TDS_X_pulse_ZF=TDS_X_pulse_ZF.*exp(-t/T2);

% Fourier transform
FDS_X_pulse_ZF = FourierTransform(TDS_X_pulse_ZF,L);
```

Ultra-low field case

```
% Vector for the time domain signal
TDS_X_pulse_ULF=zeros(K,1);

% Initialization of the density mat
rho=rho_afterpuse;
for k=1:K
    % Computes the expectation value of the field along z
    TDS_X_pulse_ULF(k)=real(trace(O*rho));

    % Propagates during dt
    rho=U_ULF*rho*U_ULF';
end

% Relaxation
TDS_X_pulse_ULF=TDS_X_pulse_ULF.*exp(-t/T2);

% Fourier transform
FDS_X_pulse_ULF = FourierTransform(TDS_X_pulse_ULF,L);
```

Plot the results

```
figure("Units","centimeters","Position",[0 0 FigureWidth FigureHeight])
subplot(1,4,1)
plot(t,TDS_X_pulse_ULF,'LineWidth',lw,'color',c2), hold on
plot(t,TDS_X_pulse_ZF,'LineWidth',lw,'color',c1), hold off
xlim([0 taq]), %xlabel('Time (s)')
ylim(YlimFID), ylabel('<{\itB}_z> (pT)')
set(gca,'fontsize',7)

subplot(1,4,[2 4])
plot(nu,FDS_X_pulse_ULF,'LineWidth',lw,'color',c2), hold on
plot(nu,FDS_X_pulse_ZF,'LineWidth',lw,'color',c1), hold off
xlim(Xlim), %xlabel('Frequency (Hz)')
set(gca,'fontsize',7)
ylim(YlimSpectra)

exportgraphics(gcf,fullfile(FigurePath, 'XA_system_adiabatic_and_pulse_X.pdf'),'ContentType','vector')
```

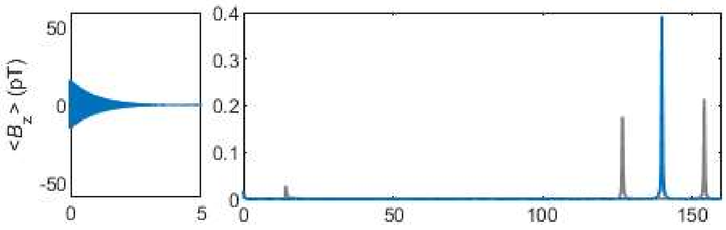

Nutation curve with pulse along x

```
% Vector containing the pulse lengths in s
tpulse=linspace(0,3000e-6,3000);

% Vector for signal as a function of the pulse length
I_X_pulse_nutation=zeros(length(tpulse),1);

parfor i=1:length(tpulse)
    % Vector for the time domain signal
    TDS_X_pulse_nutation=zeros(K,1);

    % Propagator of the pulse Hamiltonian
    Upulse=expm(-1i*tpulse(i)*HZ(Bpulse,0,0));

    % Initialization of the density mat
    rho=Upulse*rho_adia*Upulse';
    for k=1:K
        % Computes the expectation value of the field along z
        TDS_X_pulse_nutation(k)=real(trace(O*rho));

        % Propagates during dt
        rho=U_ZF*rho*U_ZF';
    end

    % Apodization - line broadening
    TDS_X_pulse_nutation=TDS_X_pulse_nutation.*exp(-t/T2);

    % Fourier transform
    FDS_X_pulse_nutation=FourierTransform(TDS_X_pulse_nutation,L);

    % Chops the relevant portion of the spectrum
    S_=FDS_X_pulse_nutation(nu>numin & nu<numax);

    % Integrates the portion of the spectrum
    I_X_pulse_nutation(i)=sum(S_);
end
```

```
Starting parallel pool (parpool) using the 'local' profile ...
Connected to the parallel pool (number of workers: 4).
```

```
figure("Units","centimeters","Position",[0 0 FigureWidth2 FigureHeight2])
plot(tpulse*1e6,I_X_pulse_nutation,'LineWidth',lw)
yline(I_sudden_drop,'k--')
ylabel('<{\itB}_z> (pT)'), %xlabel('Pulse length (\mus)')
ylim([-20 20])
set(gca,'fontsize',7)

exportgraphics(gcf,fullfile(FigurePath, 'XA_ZULF_nutation_curve_X.pdf'),'ContentType','vector')
```

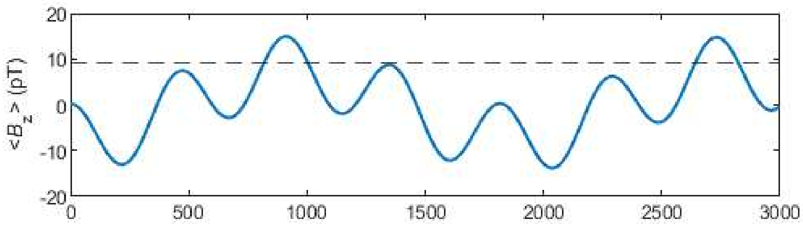


```
disp(['Signal ratio with respect to sudden drop: ' num2str(max(I_X_pulse_nutation)/I_sudden_drop)])
```

```
Signal ratio with respect to sudden drop: 1.64
```

Nutation curve with pulse along z

```
% Vector containing the pulse lengths in s
tpulse=linspace(0,3000e-6,3000);

% Vector for signal as a function of the pulse length
I_Z_pulse_nutation=zeros(length(tpulse),1);
```

```
parfor i=1:length(tpulse)
    % Vector for the time domain signal
    TDS_Z_pulse_nutation=zeros(K,1);

    % Propagator of the pulse Hamiltonian
    Upulse=expm(-1i*tpulse(i)*HZ(0,0,Bpulse));

    % Initialization of the density mat
    rho=Upulse*rho_adia*Upulse';
    for k=1:K
        % Computes the expectation value of the field along z
        TDS_Z_pulse_nutation(k)=real(trace(O*rho));

        % Propagates during dt
        rho=U_ZF*rho*U_ZF';
    end

    % Apodization - line broadening
    TDS_Z_pulse_nutation=TDS_Z_pulse_nutation.*exp(-t/T2);

    % Fourier transform
    phase=pi/2;
    FDS_Z_pulse_nutation=FourierTransform(TDS_Z_pulse_nutation*exp(1i*phase),L);

    % Chops the relevant portion of the spectrum
    S_=FDS_Z_pulse_nutation(nu>numin & nu<numax);

    % Integrates the portion of the spectrum
    I_Z_pulse_nutation(i)=sum(S_);
end

figure("Units","centimeters","Position",[0 0 FigureWidth2 FigureHeight2])
plot(tpulse*1e6,I_Z_pulse_nutation,'LineWidth',lw)
yline(I_sudden_drop,'k--')
ylabel('<{\itB}_z> (pT)')
%xlabel('Pulse length (\mus)')
ylim([-20 20])
set(gca,'fontsize',7)

exportgraphics(gcf,fullfile(FigurePath, 'XA_ZULF_nutation_curve_Z.pdf'),'ContentType','vector')
```

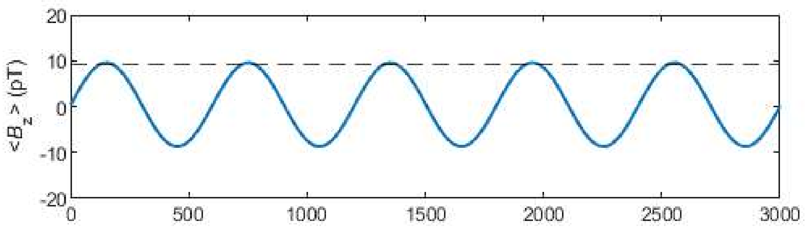

Functions

```
function S=FourierTransform(I,N)
    % Converts to column vector
    I=I(:);

    % Window function to avoid baseline shift
    I=[I(1); 2*I(2:end-1); I(end)];

    % Fourier transform
    S = real(fft(I,N))/N;

    % Remove negative frequencies
    S=2*S(1:N/2);
end
```

Supplement 5: Simulation of NMR spectra at zero and ultra-low field - Case of XAn spin system

Supplementary material to "Simulation of NMR spectra at zero- and ultra-low field from A to Z – a tribute to Prof. Konstantin L'vovich Ivanov" https://doi.org/10.5194/mr-2022-18

Q. Stern and K. Sheberstov

This script shows how to simulate ZULF spectra for an XAn spin system. It was used to generate Figure 6 of the paper.

Figure path and plot options

```
clear all
FigurePath='C:\Users\quent\Documents\Projects\Tutorial paper on ZULF\Figures\';
c1=[0    0.4470    0.7410];
c2=[1 1 1]*0.5;
lw=1;
```

General paramaters and operators

Spin system paramaters

```
JIS=140;            % J-coupling between I and S in Hz
JSS=10;             % J-coupling between S spins in Hz
gI= 67.262e6;       % Gyromagnetic ration of spin I (13C) in rad.s-1.T-1
gS=276.513e6;       % Gyromagnetic ration of spin S (1H) in rad.s-1.T-1
```

Experimental conditions

```
Bpol=2;             % Field for prepolarization in T
T=298;              % Temperature of prepolarization in K
Bx=0.5e-6;          % Magnetic field along x for ultra-low field experiments in T
Bpulse=50e-6;       % Magnetic field along x during DC pulses in T
NA=6.02e23;         % Avogadro's number in mol-1
C=27;               % Molecule concentration in mol.L-1
V=100e-6;           % Sample volume in L
r=0.01;             % Distance between the center of the sample and the center of the detector in m
```

Physical constants

```
hbar=1.05457e-34;   % Reduced Planck constant in J.s
kB=1.3806485e-23;   % Boltzmann constant J.K-1
mu0=4*pi*1e-7;      % Permeability of free space dived in T.m.A-1
```

Parameters for propagation

```
taq=5;              % Propagation/acquisition time in s
K=2^13;             % Number of points in the FID
L=2^16;             % Number of points in the FID with zero filling
T2=1;               % Coherence time constant in s
dt=taq/K;           % Time interval for propagation in s
t=(0:K-1)'/K*taq;   % Time axis in s
```

Frequency axis

```
f=(K-1)/taq;        % Sampling frequency in Hz
nu=(0:L/2-1)/L*f;   % Frequency axis in Hz
```

Initial polarization

Polarization of spin I at equilibrium in the prepolarizing magnet calculated using Boltzman distribution

```
PI=tanh(hbar*Bpol*gI/kB/T/2);
```

Polarization of spin S at equilibrium in the prepolarizing magnet calculated using Boltzman distribution

```
PS=tanh(hbar*Bpol*gS/kB/T/2);
```

Cells to store the FID and spectra

Maximum number of X spins

```
N=5;
```

Cells to stores the time domain signals

```
TDS_ZF=cell(N,1);
TDS_ULF=cell(N,1);
```

Cells to store the frequency domain signals

```
FDS_ZF=cell(N,1);
FDS_ULF=cell(N,1);
```

Basis operators in single-spin Hilbert space

Pauli matrices

```
e =[1 0;  0 1];
sx=[0 1;  1 0];
sy=[0 -1i;1i 0];
sz=[1 0;  0 -1];
```

Angular momentum operators in single spin space

```
Ix_=sx/2;
Iy_=sy/2;
Iz_=sz/2;
E=eye(2);
```

Number of molecules in the sample

```
N_molecules=NA*C*V;
```

Loops over the number of X spins

```
parfor n=1:N
    % Total number of spins of the AXn spins system
    m=1+n;

    % Constructs the operators for m-dimensional Hilbert space
    Ix=cell(m,1);
    Iy=cell(m,1);
    Iz=cell(m,1);
    for i=1:m
        Ix{i}=1; Iy{i}=1; Iz{i}=1;
        for j=1:m
            if i==j
                Ix{i}=kron(Ix{i},Ix_);
                Iy{i}=kron(Iy{i},Iy_);
                Iz{i}=kron(Iz{i},Iz_);
            else
                Ix{i}=kron(Ix{i},E);
                Iy{i}=kron(Iy{i},E);
                Iz{i}=kron(Iz{i},E);
            end
        end
    end

    % Constructs the Zeeman Hamiltonian
    HZ=@(Bx, By, Bz) -gI*(Bx*Ix{1}+By*Iy{1}+Bz*Iz{1});
```

```
for i=2:m
    HZ=@(Bx, By, Bz) HZ(Bx, By, Bz) -gS*(Bx*Ix{i}+By*Iy{i}+Bz*Iz{i});
end

% Constructs the J-Hamiltonian
HJ=zeros(2^m);
for i=2:m
    % AX couplings
    HJ=HJ +2*pi*JIS*(Ix{1}*Ix{i}+Iy{1}*Iy{i}+Iz{1}*Iz{i});

    % XX couplings
    for j=i+1:m
        HJ=HJ +2*pi*JSS*(Ix{i}*Ix{j}+Iy{i}*Iy{j}+Iz{i}*Iz{j});
    end
end

%  Constructs the density matrix
rho0=eye(2)/2+PI*Iz_;
for i=2:m
    rho0=kron(rho0,eye(2)/2+PS*Iz_);
end

% Removes the identity
rho0=rho0-eye(2^m)/2^m;

% Constructs the propagators at zero and ultra-low field
U_ZF =expm(-1i*dt*HJ);
U_ULF=expm(-1i*dt*(HJ+HZ(Bx,0,0)));

% Constructs the observable operator in pT
O=gI*Iz{1};
for i=2:m
    O=O+gS*Iz{i};
end

% Conversion to pT
O=N_molecules*hbar*1e12*mu0/2/pi/r^3*O;

% Creates empty vectors to store the time domain signals
TDS_ZF{n}= zeros(length(t),1);
TDS_ULF{n}=zeros(length(t),1);

% Propagation loop for the ZF case
% Initialization of the density matrix
rho=rho0;
for k=1:K
    % Computes the expectation value of the field along z
    TDS_ZF{n}(k)=TDS_ZF{n}(k)+real(trace(O*rho));

    % Propagates during dt
    rho=U_ZF*rho*U_ZF';
end

% Propagation loop for the ULF case
rho=rho0;
for k=1:K
    % Computes the expectation value of the field along z
    TDS_ULF{n}(k)=TDS_ULF{n}(k)+real(trace(O*rho));

    % Propagates during dt
    rho=U_ULF*rho*U_ULF';
end

% Apodization function - line broadening
TDS_ZF{n} =TDS_ZF{n} .*exp(-t/T2);
```

```
    TDS_ULF{n}=TDS_ULF{n}.*exp(-t/T2);

    % Fourier transform
    FDS_ZF{n}  = FourierTransform(TDS_ZF{n},L);
    FDS_ULF{n} = FourierTransform(TDS_ULF{n},L);
end
```

Plots the results

```
figure("Units","centimeters","Position",[0 0 18 19])
for i=1:N
    subplot(N,5,1+(i-1)*5)
    plot(nu, real(FDS_ULF{i}),'linewidth',lw,'color',c2), hold on
    plot(nu, real(FDS_ZF{i}), 'linewidth',lw,'color',c1), hold off
    xlim([0 35]), ylim([0 2.4])
    ylabel('<{\itB}_z> (pT)')
    xticks([0 10 20 30])
    set(gca,'fontsize',7)

    if i==N
        xlabel('Frequency (Hz)')
    end

    subplot(N,5,(i-1)*5+(2:5))
    plot(nu, real(FDS_ULF{i}),'linewidth',lw,'color',c2), hold on
    plot(nu, real(FDS_ZF{i}), 'linewidth',lw,'color',c1), hold off
    xlim([100 450]), ylim([0 0.5])
    set(gca,'fontsize',7)

    if i==N
        xlabel('Frequency (Hz)')
    end
end

exportgraphics(gcf,fullfile(FigurePath, 'XAn_system_spectra.pdf'),'ContentType','vector')
```

```
Warning: Vectorized content might take a long time to create, or it might contain unexpected results. Set 'Co[illegible]
```

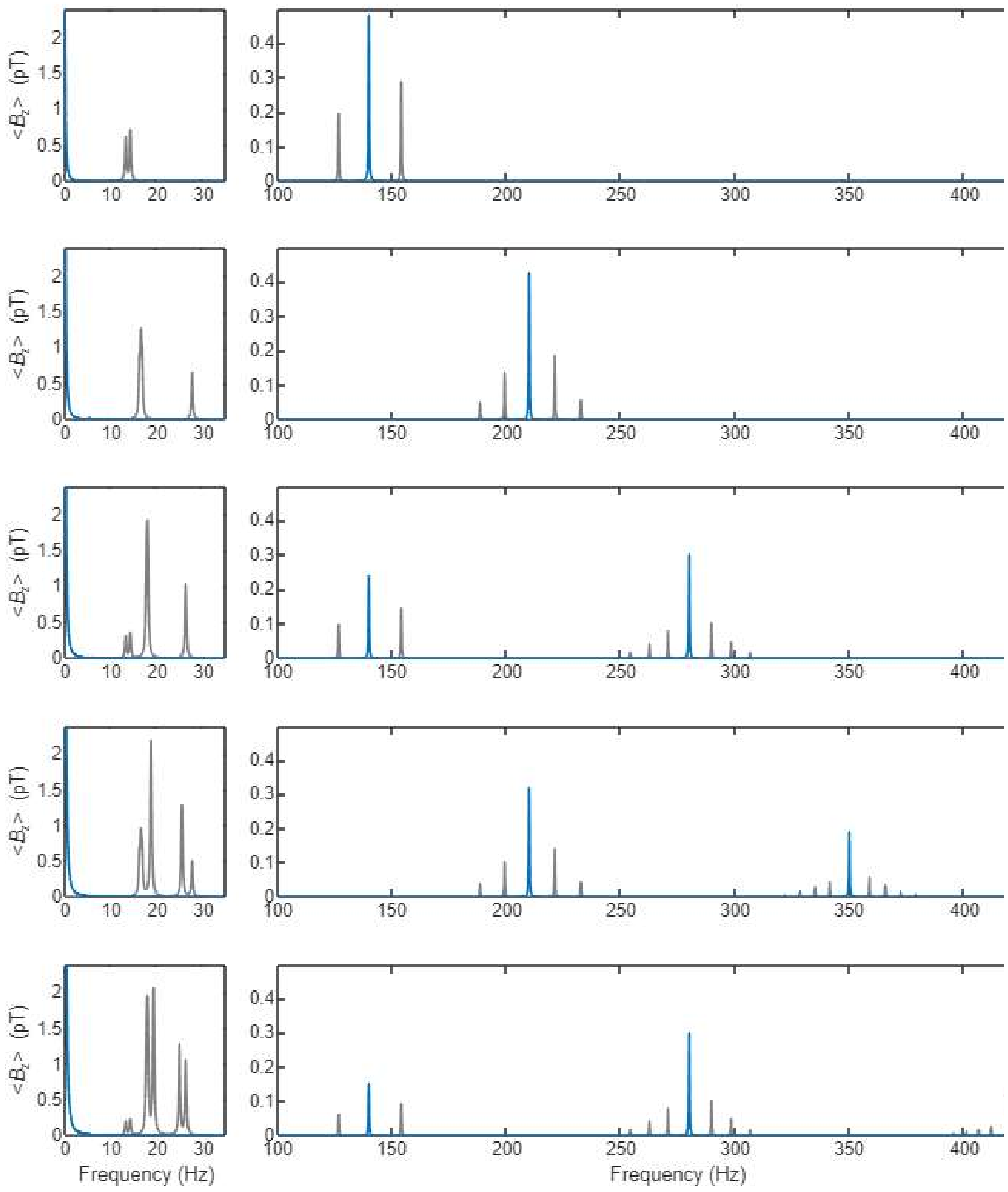

Functions

```
function S=FourierTransform(I,N)
    % Converts to column vector
    I=I(:);

    % Window function to avoid baseline shift
    I=[I(1); 2*I(2:end-1); I(end)];

    % Fourier transform
    S = real(fft(I,N))/N;

    % Remove negative frequencies
    S=2*S(1:N/2);
end
```

Supplement 6 - Analytical expressions for the eigenenergies and transition amplitudes of XA_n spin systems at zero field

Supplementary material to “Simulation of NMR spectra at zero- and ultra-low field from A to Z – a tribute to Prof. Konstantin L’vovich Ivanov” https://doi.org/10.5194/mr-2022-18

Q. Stern and K. Sheberstov

PDF and .nb Mathematica files are provided, code lines are shown in black, whereas comments are shown in blue .

Initialization of general functions

```
(*We first introduce two general functions that are used to calculate
 the eigenstates and eigenenergies for an arbitrary XAn system. After
 that we implement these functions for the cases XA, XA2, and XA3*)

SpinSystemInitialization[nspinsI_] := Module[{nstates, F, FA, FAPrevious, tmp, i, j},

  (*This function sets the spin system to XAn. First,
  it calculates the dimension of the Hilbert space. Second,
  it calculates all possible values of the total spin FA,
  and then of the total spin F. Additionally, it prints out how the list of FA values
   changes recursevily upon (n-1) summation steps in the manner of Figure 7.*)

  nstates = (2 * 1/2 + 1)^nspinsI * (2 * 1/2 + 1)^1;
  (*General expression for the Hilbert dimension of n spins I is given by (2I+1)^n*)

  FAmax = 0;
  FA = {1/2};
  (*FA is a list containing all possible values of the total spin of An spin
    subsystem. It is calculated recursively implementing Eq. 45 of the text -
   for each (n-1) summation of spins-1/2 *)
  If[nspinsI > 1,
   For[i = 1, i ≤ nspinsI - 1, i++,
    FAPrevious = FA;
    tmp = 1;
    For[j = 1, j ≤ Length[FAPrevious], j++,

     Which[FAPrevious[[j]] == 0,
      FA[[tmp]] = 1/2;
```

```
      Print[FA];
      tmp = tmp + 1,

      FAPrevious[[j]] ≠ 0,

      FA[[tmp]] = FAPrevious[[j]] + 1/2;
      tmp = tmp + 1;
      FA = Insert[FA, FAPrevious[[j]] - 1/2, tmp];
      tmp = tmp + 1;
      Print[FA]

     ]
    ]
   ]
  ];

(*F is a list containing all possible values of the total spin
  of the full XAn system. The cycle below also updates the FA list -
 because not all combinations of (F,FA) are possible. It is calculated
  similary as above by using Eq. 45 of the text. In this case,
just one summation is needed for FA of spin X*)
FAPrevious = FA;
F = FA;
tmp = 1;
For[j = 1, j ≤ Length[FAPrevious], j++,
 Which[FAPrevious[[j]] == 0,
  F[[tmp]] = 1/2;
  FA[[tmp]] = 0;
  tmp = tmp + 1,

  FAPrevious[[j]] ≠ 0,
  F[[tmp]] = FAPrevious[[j]] + 1/2;
  FA[[tmp]] = FAPrevious[[j]];

  tmp = tmp + 1;
  F = Insert[F, FAPrevious[[j]] - 1/2, tmp];
  FA = Insert[FA, FAPrevious[[j]], tmp];
  tmp = tmp + 1;
 ]
];
```

```
  {nstates, FA, F}

 ]

EnergyFmF[F_, FA_, Ispin_, Sspin_, nspins_, JAX_, JAA_] := Module[{E},
  (*This function calculates eigenenergy of a the XAn
   spin system at zero field using equation 54 of the main text*)
  E = JAX/2 (F (F + 1) - Sspin (Sspin + 1) - FA (FA + 1)) +
     JAA/2 (FA (FA + 1) - (nspins - 1) Ispin (Ispin + 1))
 ]
```

XA system

```
(*We start with the XA spin system. The spin system is constructed using the
 SpinSystemInitialization[] function. It calculates the number of spin states,
the list of all possible values for the FA quantum number and
 the list of all possible values for the F quantum number*)
```

In[∘]:=
```
nspinsA = 1;
nspins = nspinsA + 1;
{nstates, FA, F} = SpinSystemInitialization[nspinsA];
```

In[∘]:=
```
nstates
FA // MatrixForm
F // MatrixForm
```

Out[∘]= 4

Out[∘]//MatrixForm=

$$\begin{pmatrix} \frac{1}{2} \\ \frac{1}{2} \end{pmatrix}$$

Out[∘]//MatrixForm=

$$\begin{pmatrix} 1 \\ 0 \end{pmatrix}$$

```
(*The above output shows that the Hilbert space has dimension 4,
FA is always 1/2 and F equals to either 1 (triplet states) or 0 (singlet state). Now
 let us calculate the eigenvalues of 4 energy levels using EnergyFmF[] function*)
```

In[∘]:=
```
EnergyFmF[F[[1]], FA[[1]], 1 / 2, 1 / 2, nspins, JAX, JAA]
```

Out[∘]= $\frac{JAX}{4}$

In[∘]:=
```
(*Above is the energy of the three degenerat tiplet
 states. This agrees with the result of Figure 8 for AX system*)
```

In[∘]:=
```
EnergyFmF[F[[2]], FA[[1]], 1 / 2, 1 / 2, nspins, JAX, JAA]
```

Out[∘]= $-\frac{3\,JAX}{4}$

In[ ]:=
```
(*Above is the energy of the singlet state. This
 agrees with the result of Figure 8 for AX system *)
```

In[ ]:=
```
(*Now let us calculate transition amplitudes using Eq. 60. First between S0 and T0. *)
```

In[ ]:=
```
F1 = 1; (*triplet states*)
F2 = 0; (*singlet state*)
mF1 = 0; (*transition invloving the T0 state*)
mF2 = 0;(*transition invloving the S0 state*)
IntensityLine =
 (Sum[Sum[ClebschGordan[{1/2, mA}, {1/2, ms}, {F1, mF1}] * ClebschGordan[{1/2, mA},
       {1/2, ms}, {F2, mF2}] * (γi mA + γs ms),
     {ms, -1/2, 1/2, 1}], {mA, -FA[[1]], FA[[1]], 1}])^2 // FullSimplify
```

ClebschGordan: ThreeJSymbol[{1/2, -1/2}, {1/2, -1/2}, {1, 0}] is not physical.

ClebschGordan: ThreeJSymbol[{1/2, -1/2}, {1/2, -1/2}, {0, 0}] is not physical.

ClebschGordan: ThreeJSymbol[{1/2, 1/2}, {1/2, 1/2}, {1, 0}] is not physical.

General: Further output of ClebschGordan::phy will be suppressed during this calculation.

Out[ ]= $\frac{1}{4}(\gamma i - \gamma s)^2$

```
(*It can be seen that transition amplitude is porportional to the difference
 between gyromagnetic ratios and vanishes in case they are equal. Now
 let us check if there is an observable transition between S0 and T+1*)
```

In[ ]:=
```
F1 = 1; (*triplet states*)
F2 = 0; (*singlet state*)
mF1 = 1; (*transition invloving the T+1 state*)
mF2 = 0;(*transition invloving the S0 state*)
IntensityLine =
 (Sum[Sum[ClebschGordan[{FA[[1]], mA}, {1/2, ms}, {F1, mF1}] * ClebschGordan[
       {FA[[1]], mA}, {1/2, ms}, {F2, mF2}] * (γi mA + γs ms),
      {ms, -1/2, 1/2, 1}], {mA, -FA[[1]], FA[[1]], 1}])^2 // FullSimplify
```

ClebschGordan: ThreeJSymbol[{1/2, -1/2}, {1/2, -1/2}, {1, -1}] is not physical.

ClebschGordan: ThreeJSymbol[{1/2, -1/2}, {1/2, -1/2}, {0, 0}] is not physical.

ClebschGordan: ThreeJSymbol[{1/2, -1/2}, {1/2, 1/2}, {1, -1}] is not physical.

General: Further output of ClebschGordan::phy will be suppressed during this calculation.

Out[ ]= 0

In[ ]:=
```
(*Intensity is zero. Same with all other possible combinations*)
```

In[ ]:=
```
F1 = 1; (*triplet states*)
F2 = 1; (*triplet state*)
mF1 = 1; (*transition invloving the T+1 state*)
mF2 = 0;(*transition invloving the T0 state*)
IntensityLine =
 (Sum[Sum[ClebschGordan[{FA[[1]], mA}, {1/2, ms}, {F1, mF1}] * ClebschGordan[
       {FA[[1]], mA}, {1/2, ms}, {F2, mF2}] * (γi mA + γs ms),
      {ms, -1/2, 1/2, 1}], {mA, -FA[[1]], FA[[1]], 1}])^2 // FullSimplify
```

ClebschGordan: ThreeJSymbol[{1/2, -1/2}, {1/2, -1/2}, {1, -1}] is not physical.

ClebschGordan: ThreeJSymbol[{1/2, -1/2}, {1/2, -1/2}, {1, 0}] is not physical.

ClebschGordan: ThreeJSymbol[{1/2, -1/2}, {1/2, 1/2}, {1, -1}] is not physical.

General: Further output of ClebschGordan::phy will be suppressed during this calculation.

Out[ ]= 0

```
(*These calculations support the conclusions about the selection
 rules in the main text and mean that for the AX system at zero
 field only one transition between the S0 and T0 states is observable
 and only if the two nuclei have different gyromagnetic ratios.*)
```

XA_2 system

```
(*Now let us consider the XA2 spin system. The same procedure is applied here*)
```

In[ ]:=
```
nspinsA = 2;
nspins = nspinsA + 1;
{nstates, FA, F} = SpinSystemInitialization[nspinsA];
```

```
{1, 0}
```

```
(*The SpinSystemInitialization[] function prints out the recusive changes of the
 list FA for each summation step. In this case, just one summation step is done,
thus one line showing that FA can be either 1 (triplet) or 0 (singlet) *)
```

In[ ]:=
```
nstates
FA // MatrixForm
F // MatrixForm
```

Out[ ]= 8

Out[ ]//MatrixForm=

$$\begin{pmatrix} 1 \\ 1 \\ 0 \end{pmatrix}$$

Out[ ]//MatrixForm=

$$\begin{pmatrix} \frac{3}{2} \\ \frac{1}{2} \\ \frac{1}{2} \end{pmatrix}$$

```
(*The Hilbert space has dimension 8,
SpinSystemInitialization[] function produces lists FA and F of the same length,
becuase not all different combinations are possible. For example,
the highest value of F=3/2 can be achieved only in case of the highest value FA=1.*)
```

In[ ]:=
```
EnergyFmF[F[[1]], FA[[1]], 1 / 2, 1 / 2, nspins, JAX, JAA]
```

Out[ ]= $\frac{JAA}{4} + \frac{JAX}{2}$

```
(*Above is the energy of the four degenerate states with F =
 3/2 and FA = 1. See Figure 8 for XA2.*)
```

In[ ]:=
```
EnergyFmF[F[[2]], FA[[2]], 1 / 2, 1 / 2, nspins, JAX, JAA]
```

Out[ ]= $\frac{JAA}{4} - JAX$

```
(*Above is the energy of the two degenerate states with F =
 1/2 and FA = 1. See Figure 8 for XA2.*)
```

In[ ]:=
```
EnergyFmF[F[[3]], FA[[3]], 1 / 2, 1 / 2, nspins, JAX, JAA]
```

Out[ ]= $-\frac{3\,JAA}{4}$

```
(*Above is the energy of the two degenerate states with F =
 1/2 and FA = 0. See Figure 8 for XA2.*)
```

```
(*Now let us calculate the transition amplitudes using Eq. 60. First, for the
 observable line between states with (F = 1/2, F_A = 1) and (F = 3/2, F_A = 1).*)

F1 = F[[1]]
(*Here F1 denotes one of the levels
 between which transition amplitude is calculated*)
F2 = F[[2]]
(*Accordingly, here F2 denotes the second level involved in the transition*)
fa = FA[[1]]
(*F_A determines over which projection numbers m_A the summation is performed. It
 was shown in the text that F_A can not change for observable transitions so it
 does not matter where it is taken from the first or from the second state. *)
mF1 = 1 / 2;
mF2 = 1 / 2;
(*For odd number n of spins A, the total spin F is always half
  integer for XA_n so that central levels would have m_F = +-1/2  *)
IntensityLine =
 (Sum[Sum[ClebschGordan[{fa, mA}, {1/2, ms}, {F1, mF1}] * ClebschGordan[{fa, mA},
       {1/2, ms}, {F2, mF2}] * (γi mA + γs ms),
      {ms, -1/2, 1/2, 1}], {mA, -fa, fa, 1}])^2 // FullSimplify
```

Out[ ]= $\frac{3}{2}$

Out[ ]= $\frac{1}{2}$

Out[ ]= 1

ClebschGordan: ThreeJSymbol[{1, −1}, $\{\frac{1}{2}, -\frac{1}{2}\}$, $\{\frac{3}{2}, -\frac{1}{2}\}$] is not physical.

ClebschGordan: ThreeJSymbol[{1, −1}, $\{\frac{1}{2}, -\frac{1}{2}\}$, $\{\frac{1}{2}, -\frac{1}{2}\}$] is not physical.

ClebschGordan: ThreeJSymbol[{1, −1}, $\{\frac{1}{2}, \frac{1}{2}\}$, $\{\frac{3}{2}, -\frac{1}{2}\}$] is not physical.

General: Further output of ClebschGordan::phy will be suppressed during this calculation.

Out[ ]= $\frac{2}{9}(\gamma i - \gamma s)^2$

```
F1 = F[[1]]
F2 = F[[2]]
fa = FA[[1]]
mF1 = -1 / 2;
mF2 = -1 / 2;
(*We repeat same as above calculation but for the negative value of mF*)
IntensityLine =
 (Sum[Sum[ClebschGordan[{fa, mA}, {1/2, ms}, {F1, mF1}] * ClebschGordan[{fa, mA},
       {1/2, ms}, {F2, mF2}] * (γi mA + γs ms),
     {ms, -1/2, 1/2, 1}], {mA, -fa, fa, 1}])^2 // FullSimplify
```

Out[]= $\frac{3}{2}$

Out[]= $\frac{1}{2}$

Out[]= 1

ClebschGordan: ThreeJSymbol[{1, −1}, $\{\frac{1}{2}, -\frac{1}{2}\}$, $\{\frac{3}{2}, \frac{1}{2}\}$] is not physical.

ClebschGordan: ThreeJSymbol[{1, −1}, $\{\frac{1}{2}, -\frac{1}{2}\}$, $\{\frac{1}{2}, \frac{1}{2}\}$] is not physical.

ClebschGordan: ThreeJSymbol[{1, 0}, $\{\frac{1}{2}, \frac{1}{2}\}$, $\{\frac{3}{2}, \frac{1}{2}\}$] is not physical.

General: Further output of ClebschGordan::phy will be suppressed during this calculation.

Out[]= $\frac{2}{9}(\gamma i - \gamma s)^2$

```
(*The above results verify that both transitions
 are observable as shown in the central part of Figure 8 in
 the main text. All other possible transitions are forbidden,
for example where mF changes it's value (which is an illustration of Eq. 61):*)
```

In[ ]:=
```
F1 = F[[1]]
F2 = F[[2]]
fa = FA[[1]]
mF1 = 1 / 2;
mF2 = -1 / 2;

IntensityLine =
 (Sum[Sum[ClebschGordan[{fa, mA}, {1/2, ms}, {F1, mF1}] * ClebschGordan[{fa, mA},
      {1/2, ms}, {F2, mF2}] * (γi mA + γs ms),
     {ms, -1/2, 1/2, 1}], {mA, -fa, fa, 1}])^2 // FullSimplify
```

Out[ ]= $\frac{3}{2}$

Out[ ]= $\frac{1}{2}$

Out[ ]= 1

ClebschGordan: ThreeJSymbol[{1, −1}, {1/2, −1/2}, {3/2, −1/2}] is not physical.

ClebschGordan: ThreeJSymbol[{1, −1}, {1/2, −1/2}, {1/2, 1/2}] is not physical.

ClebschGordan: ThreeJSymbol[{1, −1}, {1/2, 1/2}, {3/2, −1/2}] is not physical.

General: Further output of ClebschGordan::phy will be suppressed during this calculation.

Out[ ]= 0

XA_3 system

```
(*Now let us consider the XA3 spin system*)
```

In[ ]:=
```
nspinsA = 3;
nspins = nspinsA + 1;
{nstates, FA, F} = SpinSystemInitialization[nspinsA];
```

{1, 0}

$\left\{\frac{3}{2}, \frac{1}{2}, 0\right\}$

$\left\{\frac{3}{2}, \frac{1}{2}, \frac{1}{2}\right\}$

```
(*In this case, two summation steps are identical to those shown in Figure 7*)
```

In[ ]:=
```
nstates
FA // MatrixForm
F // MatrixForm
```

Out[ ]= 16

Out[ ]//MatrixForm=

$$\begin{pmatrix} \frac{3}{2} \\ \frac{3}{2} \\ \frac{1}{2} \\ \frac{1}{2} \\ \frac{1}{2} \\ \frac{1}{2} \end{pmatrix}$$

Out[ ]//MatrixForm=

$$\begin{pmatrix} 2 \\ 1 \\ 1 \\ 0 \\ 1 \\ 0 \end{pmatrix}$$

(*The Hilbert space has dimension 16. The highest value of F= 2 can be achieved only in case of the highest value F_A=3/2 etc.*)

In[ ]:=
```
EnergyFmF[F⟦1⟧, FA⟦1⟧, 1 / 2, 1 / 2, nspins, JAX, JAA]
EnergyFmF[F⟦2⟧, FA⟦2⟧, 1 / 2, 1 / 2, nspins, JAX, JAA]
EnergyFmF[F⟦3⟧, FA⟦3⟧, 1 / 2, 1 / 2, nspins, JAX, JAA]
EnergyFmF[F⟦4⟧, FA⟦4⟧, 1 / 2, 1 / 2, nspins, JAX, JAA]
EnergyFmF[F⟦5⟧, FA⟦5⟧, 1 / 2, 1 / 2, nspins, JAX, JAA]
EnergyFmF[F⟦6⟧, FA⟦6⟧, 1 / 2, 1 / 2, nspins, JAX, JAA]
```

Out[ ]= $\frac{3\,JAA}{4} + \frac{3\,JAX}{4}$

Out[ ]= $\frac{3\,JAA}{4} - \frac{5\,JAX}{4}$

Out[ ]= $-\frac{3\,JAA}{4} + \frac{JAX}{4}$

Out[ ]= $-\frac{3\,JAA}{4} - \frac{3\,JAX}{4}$

Out[ ]= $-\frac{3\,JAA}{4} + \frac{JAX}{4}$

Out[ ]= $-\frac{3\,JAA}{4} - \frac{3\,JAX}{4}$

(*Above the energies for all possible manifolds of (F,F_A) states are shown, they can be compared to the left section of Figure 8.*)

(*Now let us calculate transition amplitudes, again using Eq. 60.*)

In[ ]:=
```
F1 = F[[1]]
F2 = F[[2]]
fa = FA[[1]]
mF1 = 0;
mF2 = 0;
IntensityLine =
 (Sum[Sum[ClebschGordan[{fa, mA}, {1/2, ms}, {F1, mF1}] * ClebschGordan[{fa, mA},
       {1/2, ms}, {F2, mF2}] * (γi mA + γs ms),
     {ms, -1/2, 1/2, 1}], {mA, -fa, fa, 1}])^2 // FullSimplify
```

Out[ ]= 2

Out[ ]= 1

Out[ ]= $\frac{3}{2}$

ClebschGordan: ThreeJSymbol[$\{\frac{3}{2}, -\frac{3}{2}\}$, $\{\frac{1}{2}, -\frac{1}{2}\}$, {2, 0}] is not physical.

ClebschGordan: ThreeJSymbol[$\{\frac{3}{2}, -\frac{3}{2}\}$, $\{\frac{1}{2}, -\frac{1}{2}\}$, {1, 0}] is not physical.

ClebschGordan: ThreeJSymbol[$\{\frac{3}{2}, -\frac{3}{2}\}$, $\{\frac{1}{2}, \frac{1}{2}\}$, {2, 0}] is not physical.

General: Further output of ClebschGordan::phy will be suppressed during this calculation.

Out[ ]= $\frac{1}{4}(\gamma i - \gamma s)^2$

In[ ]:= (*Intensity of the $2J_{AX}$ line is nonzero*)

In[ ]:=
```
F1 = F[[3]]
F2 = F[[4]]
fa = FA[[3]]
mF1 = 0;
mF2 = 0;
IntensityLine =
 (Sum[Sum[ClebschGordan[{fa, mA}, {1/2, ms}, {F1, mF1}] * ClebschGordan[{fa, mA},
       {1/2, ms}, {F2, mF2}] * (γi mA + γs ms),
     {ms, -1/2, 1/2, 1}], {mA, -fa, fa, 1}])^2 // FullSimplify
```

Out[ ]= 1

Out[ ]= 0

Out[ ]= $\frac{1}{2}$

ClebschGordan: ThreeJSymbol[$\{\frac{1}{2}, -\frac{1}{2}\}$, $\{\frac{1}{2}, -\frac{1}{2}\}$, {1, 0}] is not physical.

ClebschGordan: ThreeJSymbol[$\{\frac{1}{2}, -\frac{1}{2}\}$, $\{\frac{1}{2}, -\frac{1}{2}\}$, {0, 0}] is not physical.

ClebschGordan: ThreeJSymbol[$\{\frac{1}{2}, \frac{1}{2}\}$, $\{\frac{1}{2}, \frac{1}{2}\}$, {1, 0}] is not physical.

General: Further output of ClebschGordan::phy will be suppressed during this calculation.

Out[ ]= $\frac{1}{4}(\gamma i - \gamma s)^2$

(*As expected, the intensity of the $1J_{AX}$ line is also nonzero. The Reader is encoraged to write a cycle for all other possible combinations of F1 and F2. However, one should be carefull when verifying transition amplitudes between the two states with different F_A. The amplitude of all such transitions is zero, but this is due to the zero value Bra.Ket product which is taken into account in the more general Eq. 58. Therefore, in order to perform a proper general simulation one should write a program that constructs all the Hilbert functions themselves and can calculate the Bra.Ket products. The limitation of eq. 60 is illustrated below for a transition that should have a zero amplitude:*)

In[ ]:=
```
F1 = F[[1]]
F2 = F[[3]]
fa = FA[[1]]
mF1 = 0;
mF2 = 0;
IntensityLine =
 (Sum[Sum[ClebschGordan[{fa, mA}, {1/2, ms}, {F1, mF1}] * ClebschGordan[{fa, mA},
       {1/2, ms}, {F2, mF2}] * (γi mA + γs ms),
      {ms, -1/2, 1/2, 1}], {mA, -fa, fa, 1}])^2 // FullSimplify
```

Out[ ]= 2

Out[ ]= 1

Out[ ]= $\frac{3}{2}$

ClebschGordan: ThreeJSymbol[{3/2, -3/2}, {1/2, -1/2}, {2, 0}] is not physical.

ClebschGordan: ThreeJSymbol[{3/2, -3/2}, {1/2, -1/2}, {1, 0}] is not physical.

ClebschGordan: ThreeJSymbol[{3/2, -3/2}, {1/2, 1/2}, {2, 0}] is not physical.

General: Further output of ClebschGordan::phy will be suppressed during this calculation.

Out[ ]= $\frac{1}{4}(\gamma i - \gamma s)^2$

```
(*The purpose of this code is educational,
it provides some simple examples of how to use Clebsch-
 Gordan coefficents to get analytical expressions for ZULF
  NMR. These acquired skills can be usefull to combine with full
  spin dynamics simulation tools. We wish you good luck with that!*)
```
